# Impact of the COVID-19 Pandemic on Older Adults: Rapid Review

**DOI:** 10.2196/26474

**Published:** 2021-04-12

**Authors:** Audrey Lebrasseur, Noémie Fortin-Bédard, Josiane Lettre, Emilie Raymond, Eve-Line Bussières, Nolwenn Lapierre, Julie Faieta, Claude Vincent, Louise Duchesne, Marie-Christine Ouellet, Eric Gagnon, André Tourigny, Marie-Ève Lamontagne, François Routhier

**Affiliations:** 1 Centre for Interdisciplinary Research in Rehabilitation and Social Integration Centre intégré universitaire de santé et de services sociaux de la Capitale-Nationale Québec, QC Canada; 2 Department of Rehabilitation Université Laval Québec, QC Canada; 3 School of Social Work and Criminology Université Laval Québec, QC Canada; 4 Department of Psychology Université du Québec à Trois-Rivières Trois-Rivières, QC Canada; 5 Department of Speech-Language Pathology Université du Québec à Trois-Rivières Trois-Rivières, QC Canada; 6 School of Psychology Université Laval Québec, QC Canada; 7 VITAM – Centre de recherche en santé durable Centre intégré universitaire de santé et de services sociaux de la Capitale-Nationale Québec, QC Canada; 8 Department of Sociology Université Laval Québec, QC Canada; 9 Department of Social and Preventive Medicine Université Laval Québec, QC Canada

**Keywords:** COVID-19, impact, rapid review, older adults, aged individuals, review

## Abstract

**Background:**

The COVID-19 pandemic has drastically changed the lives of countless members of the general population. Older adults are known to experience loneliness, age discrimination, and excessive worry. It is therefore reasonable to anticipate that they would experience greater negative outcomes related to the COVID-19 pandemic given their increased isolation and risk for complications than younger adults.

**Objective:**

This study aims to synthesize the existing research on the impact of the COVID-19 pandemic, and associated isolation and protective measures, on older adults. The secondary objective is to investigate the impact of the COVID-19 pandemic, and associated isolation and protective measures, on older adults with Alzheimer disease and related dementias.

**Methods:**

A rapid review of the published literature was conducted on October 6, 2020, through a search of 6 online databases to synthesize results from published original studies regarding the impact of the COVID-19 pandemic on older adults. The Human Development Model conceptual framework–Disability Creation Process was used to describe and understand interactions between personal factors, environmental factors, and life habits. Methods and results are reported following the Preferred Reporting Items for Systematic Reviews and Meta-analyses Statement.

**Results:**

A total of 135 records were included from the initial search strategy of 13,452 individual studies. Of these, 113 (83.7%) studies were determined to be of level 4 according to the levels of evidence classification by the Centre for Evidence-Based Medicine. The presence of psychological symptoms, exacerbation of ageism, and physical deterioration of aged populations were reported in the included studies. Decreased social life and fewer in-person social interactions reported during the COVID-19 pandemic were occasionally associated with reduced quality of life and increased depression. Difficulties accessing services, sleep disturbances, and a reduction of physical activity were also noted.

**Conclusions:**

Our results highlight the need for adequate isolation and protective measures. Older adults represent a heterogeneous group, which could explain the contradictory results found in the literature. Individual, organizational, and institutional strategies should be established to ensure that older adults are able to maintain social contacts, preserve family ties, and maintain the ability to give or receive help during the current pandemic. Future studies should focus on specific consequences and needs of more at-risk older adults to ensure their inclusion, both in public health recommendations and considerations made by policy makers.

## Introduction

### Background

Since the end of 2019, the SARS-CoV-2 outbreak has resulted in more than 71 million cases worldwide, as of December 16, 2020 [1]. Isolation and protective measures have been established by governments to varying extents around the world in order to mitigate the spread of the virus. These measures include physical distancing, use of face masks, handwashing, stay-at-home policies, and restrictions on social gatherings [2,3]. As a result, the general population has experienced drastic changes in day-to-day life [4]; high COVID-19–related fear [5]; and numerous psychological outcomes such as depression [6], increased sleep problems [7], and financial worries [8].

However, the extent to which the effects of COVID-19 reported by the general population are experienced by the aging population is not well documented. Isolation and protective measures are crucial for the aging population, who are at greater risk of COVID-19–related death [9]. However, isolation and protective measures may also amplify issues that are already present in older adults, such as loneliness, age discrimination, and excessive worrying [10-12]. Considering that physical distancing inevitably leads to some degree of social isolation, speculation towards the pernicious impact of physical distancing on the mental health, daily activities [12], and cognitive decline of older adults [11] is warranted. The COVID-19 pandemic may also amplify age discrimination by negatively impacting access to information, health care services, and support to informal caregivers and familial advocates [13,14].

According to the existing literature, although many older adults are now online [15,16], the majority still need assistance when using digital technologies and to access and assess information [17]. Furthermore, most vulnerable older adults do not have access to web resources or the required digital skills and knowledge for its use to be satisfying and efficient [15,18]. Digital technology is thus insufficient to reach vulnerable populations such as older adults [19].

The fear of contracting the virus could be an additional source of concern for this population, thus contributing to the overall anxiety—a mental health outcome already known to negatively affect the quality of life in older adults [10]. Thus, it is possible that the immediate and long-term effects of the COVID-19 pandemic are heightened for older adults as compared to other age demographics.

Since the beginning of the pandemic, there has been substantial concern surrounding older adults living in nursing home [20]. The percentage of nursing home residents with Alzheimer disease or other types of dementia is significant, reported to range between 45% and 75% [21-23]. It is possible that people with Alzheimer disease or other dementias are experiencing greater negative outcomes related to the COVID-19 pandemic.

A better understanding of the unique experiences of older adults during the pandemic is needed in order for governing bodies and health care providers to design adequate policies [13] and services as we advance. Therefore, data specific to the needs of older adults within the context of the present COVID-19 pandemic are urgently needed.

### Objectives

The aim of this study is to synthesize the existing research on the impact of the COVID-19 pandemic, and associated isolation and protective measures, on older adults. Furthermore, we aim to investigate the impact of the COVID-19 pandemic, and associated isolation and protective measures, on older adults with Alzheimer disease and related dementias.

## Methods

### Protocol

Given the urgent need for adequate information, a rapid review protocol was chosen. This type of review is conducted using an accelerated systematic review method, which limits certain aspects of the methodology in order to provide evidence within a policy maker’s timeframe [24,25]. This approach aligns with the available guidance for Cochrane Rapid Review Methods Group [25] and with the Practical Guide for Rapid Reviews to Strengthen Health Policy and Systems [26]. Methods and results are reported following the PRISMA (Preferred Reporting Items for Systematic Reviews and Meta-analyses) Statement [27]. The protocol for the present review was registered within the PROSPERO database (ID: CRD42020201814).

### Conceptual Framework

The Human Development Model–Disability Creation Process (HDM-DCP) conceptual framework was used to describe and understand interactions between personal factors, environmental factors, and life habits [28]. The HDM-DCP model acknowledges the impact of the environment and the person on the execution of life habits. Personal factors include identity (facilitator or obstacle), organic systems (integrity or impairment), and capabilities (ability or disability). Environmental factors are stratified into societal (facilitator or obstacle), community (facilitator or obstacle), and personal (facilitator or obstacle) levels. Life habits consist of daily activities (social participation situation or disabling situation) and social roles (social participation situation or disabling situation). Each of these elements can be seen as a protective factor or as a risk factor for the individual. The HDM-DCP framework allows observation of changes in these domains over a period of time (eg, the span of the COVID-19 pandemic). The framework puts into evidence social participation and social contacts, both of which may be greatly affected by pandemic-related isolation and protective measures.

### Literature Search

Search strategies were developed by two authors (AL and NFB) and reviewed by two other authors (FR and ML). These strategies centered around three concepts: “COVID-19,” “older adults,” and “impact.” The concept “COVID-19” was used to restrict the obtained results to those related to the present pandemic. According to the World Health Organization [29], older adults include people of 60 years of age and older. Therefore, in this study, the concept “older adults” included people aged 60 years and older, without excluding any diagnoses or conditions. The concept “impact” encompasses all three domains of the HDM-DCP model (ie, personal factors, environmental factors, and life habits) [28]. “Impact” variables can be reported by an individual, caregivers, family members, or health care workers, and may vary in the way that they are experienced or perceived. The following databases were used: MEDLINE via PUBMED; Embase, PsycINFO, and PsycARTICLES via Psycnet; and CINAHL and Ageline via EBSCOhost. The searches were conducted on October 6, 2020. See Multimedia Appendix 1 for detailed search strategies used for each database.

### Eligibility Criteria

The Population, Exposure, Comparator, and Outcomes (PECO) framework was used to develop the eligibility criteria used for the purposes of this review (see Table 1) [30]. Eligibility criteria were defined as follows: (1) peer-reviewed original papers with data related to our research question (opinion papers, reviews, methodological articles, preprints, and unpublished documents were excluded); (2) publication dates limited to 2019 and 2020, as the COVID-19 outbreak was first reported in 2019; (3) papers available in English or French; and (4) participants 60 years of age and older with any diagnosis except for COVID-19 survivors. The fourth criterion was applied in order to differentiate the effect of the pandemic from the physiological and health-related outcomes associated with a COVID-19 diagnosis. Furthermore, only papers that specified in the abstract the inclusion of older adults in the study were included. Outcomes that did not fit into the domains of the HDM-DCP framework (eg, knowledge about the spread of the disease) were excluded. 

**Table 1:** Population, Exposure, Comparator, and Outcomes inclusion criteria.

Component	Description
Population (P)	People aged 60 years and older, excluding COVID-19 survivors
Exposure (E)	COVID-19 and its associated isolation and protective measures
Comparator (C)	Other age groups, before the pandemic, or none
Outcomes (O)	Personal factors such as identity factors (facilitator or obstacle), organic systems (integrity or impairment), and capabilities (ability or disability) Environmental factors such as societal (facilitator or obstacle), community (facilitator or obstacle), and personal (facilitator or obstacle) levels Life habits such as daily activities (social participation situation or disabling situation) and social roles (social participation situation or disabling situation)

### Study Selection and Data Extraction

Data retrieved from the databases were exported to Covidence [31]. Two reviewers independently screened the titles and abstracts of the obtained records. These reviewers then read the full text of the selected papers and determined whether they should be included. Any disagreement was resolved via consensus. Next, a single reviewer completed data extraction, which was then verified by another reviewer. The following variables were extracted: title, year of publication, country, study design, objectives, participant characteristics (eg, diagnosis and age), and outcomes. The references of the included papers were screened by the reviewers (one reviewer per study), and the titles and abstracts of additional papers were screened if relevant.

### Level of Evidence Appraisal and Data Synthesis

Two reviewers established the level of evidence for each selected study, based on the levels of evidence classification of the Centre for Evidence-Based Medicine [32]. Due to the limited turnaround time, no risk of bias assessment was performed. A narrative approach consistent with the data synthesis of a rapid review [24] was used.

## Results

### Literature Search

**Figure 1:**
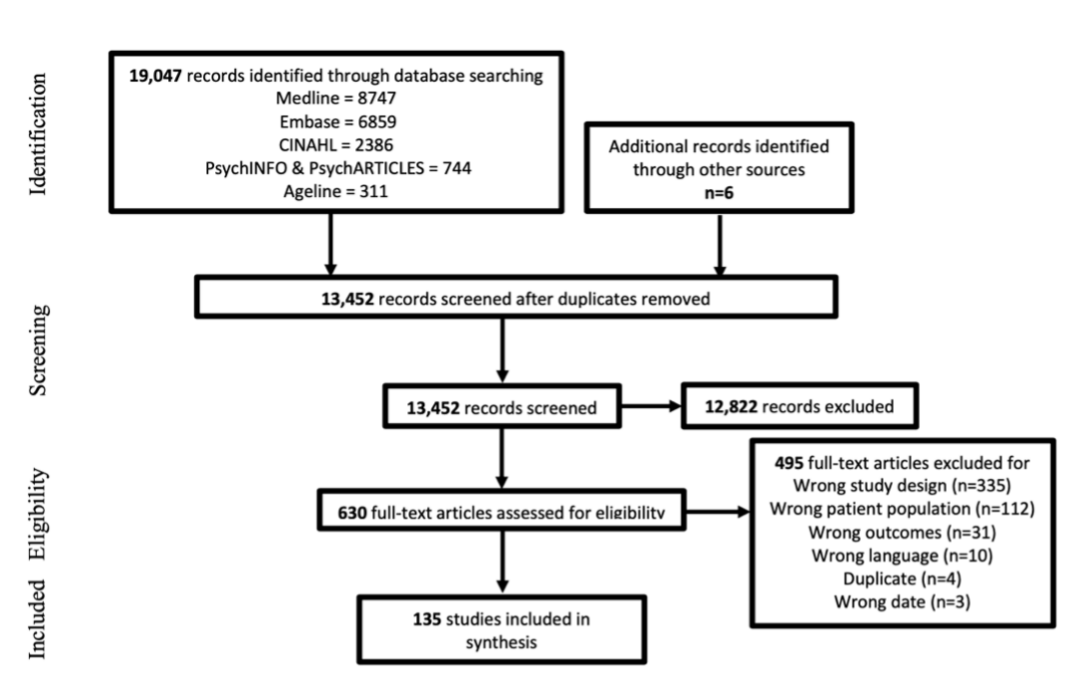
PRISMA (Preferred Reporting Items for Systematic Reviews and Meta-analyses) flow diagram.

The search strategy identified 19,053 records. A total of 13,452 records remained after duplicates (n=5601) were removed. Upon title and abstract screening, the number of papers reduced to 630, after the exclusion of 12,822 records. Thereafter, full-text screening resulted in a final inclusion of 135 records (Figure 1), following the exclusion of 495 others for various reasons.

### Characteristics of Included Studies

The selected records and their corresponding levels of evidence are shown in Table S1 of Multimedia Appendix 2. In all, 113 of 135 (83.7%) studies were determined to be of level 4 according to the Centre for Evidence-Based Medicine–Levels of Evidence [32] (transversal data collection), 20 (14.8%) studies were determined to be of level 2b (longitudinal studies), and 2 (1.5%) studies were of level 2b and level 4 (mixed study designs).

Of the 135 studies included, 40 (29.7%) studies included only older adults (≥60 years old) whereas 95 (70.3%) compared various age groups. Moreover, 15 (11.1%) studies included persons with specific conditions such as Alzheimer disease [33-35], Parkinson disease [36], frontotemporal lobar degeneration [37], severe cognitive impairments [38], ovarian cancer [39], gynecological cancer [40], patients with cancer actively treated with systemic therapy [41], pre-existing depression [42], chronic conditions [43], long-term respiratory conditions [44], migraine [45], epilepsy [46], and visual impairments [47]. A total of 29 (21.5%) studies were conducted in North America [40,42,43,48-73], 14 (10.3%) in China [46,74-86], 61 (45.1%) in Europe [33-38,41,44,87-139], 3 (2.2%) in Japan [140-142], 4 (3.0%) in Israel [143-146], 4 (3.0%) in Brazil [147-150], 4 (3.0%) in Australia [151-154], 2 (1.5%) in India [155,156], 1 (0.7%) in Malaysia [157], 2 (1.5%) in Kuwait [45,158], 1 (0.7%) in Saudi Arabia [159], 3 (2.2%) in Argentina [160-162], 1 (0.7%) in Cameroon [163], 1 (0.7%) in Russia [164], 1 (0.7%) in Ghana [47], 1 (0.7% in Cyprus [165], and 3 (2.2%) in multiple countries [39,166,167].

### Outcomes of Included Studies

#### Personal Factors

Older adults reported a presence or worsening of psychological symptoms, and greater loneliness because of pandemic-related social isolation [33,38,47,49,51,52,56,57,62,63,77,78, 84,89,90,99,105,114,117,128,135,139,140,148,156,160]. Compared to younger age, older age (ie, ≥60 years) was, however, associated with fewer psychological symptoms [39,44,50,54,57,59,64-67,74,77,89,95,97,98,101,107,109,111,114,116, 120,121,124,125,127,136,138,147,151,153,157,158,161,162,165], lower loneliness [92,95,104,130,140,159], and better mental health and well-being [95,106,126,157,161,162]. Older adults were also shown to be better at regulating their emotions and coping with stressful events [44,61,68]. In contrast, 6 (4.4%) studies reported that older adults had more severe psychological symptoms than participants of other age groups [83,85,86,96,118,156], and some studies noted no psychological symptoms for most participants [42,79,119,165].

Several variables were associated with poor psychological health and well-being, including living alone [117], decreased social interactions [88], feeling close to death, high levels of COVID-19–related health worries [145], stress [70], health concerns and ageism [143], not having cognitive impairments [38], and male status [78]. In contrast, religious faith, exercise, self-care, and time spent in nature were associated with positive psychological well-being [70].

Various worries surrounding the current pandemic were reported in these studies [36,49,105,107,110,155]. For instance, older adults were more worried about COVID-19 [68,75,96,118,167], whereas younger individuals were more concerned about the risks related to social isolation [164]. Older adults were less concerned for their emotional well-being, work goals, and finances [65], and they perceived they had lower chances of “running out of money” [53]. However, more worries about financial difficulties were reported in another study [164]. Older adults perceived the risks of COVID-19 (in comparison to that of the flu) to be higher [48,138], but aged men were less worried about COVID-19 (eg, contracting the virus, dying due to COVID-19, or disruptions to lifestyle) than their younger counterparts [48]. Their concerns were focused on others rather than themselves [144]. Anxiety associated with cancer was lower in older adults than in younger adults [41]. Expectations (eg, income decline, duration and long-term impact of COVID-19) were associated with an experience of stress, which was further associated with other negative effects [69]. Finally, the passage of time during the pandemic was found to be slower for older adults [115].

Regarding the impairment of organic systems, higher age was associated with poorer health status [80] and in some cases, a decline of functional status [163]. Decreases in mobility, functionality, vitality, and physical conditions were also noted [36,148]. An aggravation in neuropsychiatric and physical symptoms was reported in individuals with Alzheimer disease, dementia, and frontotemporal lobar degeneration, as well as in nursing home residents [34,35,37,93,139]. An exacerbation of migraine days and severity was observed among individuals with a migraine diagnosis [45]. One out of six older patients with epilepsy experienced increased seizures, but this frequency increased considerably among younger people with epilepsy [46].

#### Environmental Factors

Decreased social life and fewer in-person social interactions observed during the pandemic were occasionally associated with reduced quality of life and increased depression [42,63,128,139]. Some individuals continued to meet their relatives almost daily [36]. Furthermore, some studies reported on the negative impacts of the pandemic for caregivers [34,35].

Older adults reported unmet personal, domestic, or social needs [128]; difficulty finding help with functional needs such as bathing [62]; insufficient personal care [139]; decreased care rendered by caregivers [47]; and reductions in social support services hours [99]. Multiple barriers to care delivery were noted during this time [166]. For instance, one study reported that older adults were more likely to miss or cancel medical appointments [129], whereas another reported the opposite [60]. One study reported that treatment delays and postponed appointments were more common among older adults [130], whereas another reported this was more commonly observed among younger people [40]. More patients missed medical appointments during the pandemic as compared to the prepandemic timepoints [84,163], and rehabilitation services were discontinued for the majority due to the quarantine [160]. Finally, as compared to the previous years, psychiatry consultations for older individuals had reduced in one study [113] but reported to have increased in another [76].

#### Life Habits

Changes in sleep habits and sleep disturbances were reported to be affected by COVID-19 [37,56,84,87,105,134,160]. Of note, some studies indicated that sleep issues were lower in older adults than in younger adults [122,134,147].

Older adults reported a lower increase in unhealthy food intake, screen use, tobacco use [149], alcohol use [149,154], and cannabis use than did younger adults [123,152], in addition to a lower rise in unhealthy lifestyle changes or drinking [131]. One study indicated that the majority of older individuals consumed a balanced diet, limited their alcohol intake, and had adequate sleep patterns [82], whereas another study reported no change in alcohol use patterns [71]. This finding was contrasted by other studies that found that older adults increased binge drinking, alcohol frequency, alcohol consumption, and cigarette smoking [56,114,132]; changed their eating habits [132,148]; ate more [56,87]; and ate more often [87]. One study reported a higher consumption of unhealthy foods among older adults, as compared with participants of other age groups [149]. Food insufficiency increased in older adults during the pandemic, but to a lesser extent than that among younger adults [73], and decreased care resulted in hunger [47].

Changes in daily routine and plans were reported in a few studies [43,52,58]; however, one study noted no changes in the performance of daily habits among older adults [102]. Behavioral changes, such as buying more food and water than usual, going out less frequently, reducing social contacts, and staying away from public places were noted in several studies [36,47,58,146]. Unemployment increased in older adults, but at a lower rate than that in other age groups [72]. Higher age was associated with fewer sexual activities [108]. Some studies reported a decrease in physical activity [56,132,141,142,149] and a decline in attendance at physical activity workshops [133]. However, studies reported contradictory results regarding physical activity among older adults during the pandemic. Indeed, it was noted that older adults had the lowest levels of physical activity among all age groups [55,100]; however, they had the smallest decrease in physical activity [100], the lowest prevalence of insufficient physical activity [81] and were less likely to have changed their physical activity levels during the pandemic [137]. Moreover, physical activity was associated with higher resilience, positive affect, and lower depressive symptoms [94,141]. Older adults were also reported to have a lesser change in unhealthy movement behaviors [150].

A study indicated that a lot of time was spent learning about COVID-19 [87], and more time was spent using social media [56], internet [144], and electronic products [150]. One study reported a higher usage of electronic products by older people [149], whereas others reported contrasting findings [74,81]. Participants felt *blessed*, *lucky*, and *fortunate* to be able to stay in contact with others through social media [91]. Variation in game use by older adults did not differ from that observed in younger populations [167]. Older adults had fewer positive work events but more remote social interactions, social networks, and outdoor activities [65]. Finally, older adults engaged in more solitary activities and in fewer in-person activities [56].

One study reported that the majority of older adult travelers were planning to travel by air in the next year [103], whereas another found that older adults were canceling out-of-town trips [58]. The COVID-19 pandemic has presented significant challenges to most older adults [58], and compliance to hygiene recommendations was seen as a psychological burden by this population [148].

## Discussion

### Principal Results

Older adults are known to experience loneliness, age discrimination, and excessive worrying [10-12]. Therefore, we initially anticipated that they would experience greater negative outcomes related to the COVID-19 pandemic. However, this hypothesis was not uniformly supported by the available literature. The findings summarized within this review suggest that older adults experienced negative outcomes related to the pandemic, but to a lesser extent than their younger counterparts. Younger adults experienced greater psychological repercussions from isolation and feeling of loneliness [168]. There was indeed a correlation between young age and poor mental health [126], higher anxiety, depression, and stress [153]. This result may be explained by the daily experience of loneliness and social isolation among older adults prior to the pandemic [12], which in turn meant that COVID-19 led to fewer changes in their daily routine as compared to employed, younger adults. Another potential explanation is the influence of certain personal factors among older adults, for example, greater resilience that is associated with more purpose in life [112], better regulation of emotions, and better coping strategies in the case of stressful events [44,61,68]. These personal factors could explain the generally better psychological response by older adults throughout the COVID-19 pandemic. Additionally, these findings may be explained by sampling methods used in the available research. In other words, the isolation measures implemented in long-term care facilities may have caused additional barriers to conducting studies with residents. In our study sample, 16 (11.8%) studies were conducted among community dwelling older adults, 3 (2.2%) included older adults living in residential care facilities, 3 (2.2%) included older adults living in one of these two locations, and 113 (83.7%) studies did not detail this sampling information. Without uniform sampling methods, it is more difficult to draw strong conclusions. Older adults may have little to no access to technology [169], such as a computer or a smartphone, which are often required to participate in web-based surveys. The most isolated individuals may be the most difficult to reach, particularly if they lack access to social media or maintain minimal presence in public and community organizations—platforms often used by researchers to contact participants. Fewer opportunities to participate in surveys may explain the relative scarcity of research on vulnerable older adult populations, such as those with dementia or Alzheimer disease, during the present pandemic. In studies that compared different age groups, the proportion of aged individuals was often very small compared to other age groups. It is possible that the older adults who participated in surveys were healthy and had access to technology and, therefore, were not the most vulnerable. This could explain why certain studies suggested that younger people were more impacted than older adults.

One study reported that anxiety symptoms in older adults were associated with ageism [143], something that the current pandemic seems to have exacerbated [170]. The COVID-19 pandemic has been characterized as an older adult problem, and social media, among other platforms, have been used by people to share ageist attitudes (eg, posts published with the hashtag “BoomerRemover”) [171-173]. Greater awareness of age discrimination is needed to reduce these behaviors. There are other potential sources of anxiety among older adults, such as being unable to access support services since the onset of the COVID-19 pandemic [99]. More research is needed to understand the impact of ageism on older adults’ well-being, as compared with other risk factors.

The impact of the pandemic on older adults can also influence their caregivers [34,35]. Indeed, family caregivers reported living with anxiety and fear [174] and having difficulty balancing caregiving challenges with their own needs [175] during this crisis. It is therefore important to consider the needs of the caregivers in future policies and in the implementation of isolation and protective measures.

The available literature offered different strategies for maintaining the well-being of older adults; these included using technology to ensure social connections, pursuing outdoor activities, and incorporating daily structure [176]. Different programs were also deployed during the pandemic with the aim of reducing social isolation through contact with a student volunteer who engaged in weekly phone calls with participants living in nursing homes [177] and a single call with participants living in long-term care facilities and in the community [178]. The use of technology to protect and improve mental health [179] and to maintain the health and independence [180] of older adults during this crisis was also discussed. The transformation of an on-site program into an online program for older populations [181] was found to be effective. Innovative programs should therefore be created with the goal of supporting vulnerable older adults and minimizing the long-term consequences and feelings of loneliness.

Physical activity should be promoted during the pandemic, especially for more at-risk individuals such as those living with chronic diseases [182]. Older adults should be guided to safe and accessible physical activity programs, selected according to the individual’s level of autonomy, mobility, frailty, and health status, to avoid deconditioning during confinement. Physical activity is associated with a better quality of life [183] and decreased symptoms of depression in older adults [50], whereas increased inactivity could accelerate their physical decline [184]. Personalized physical activity programs with monitoring should therefore be made more accessible to this population to minimize deconditioning and help older adults maintain their physical and mental health, while ensuring their safety.

The secondary impacts of COVID-19 should be considered by governing bodies and institutions when taking action and making decisions about health care access and public health measures, both during the current pandemic and for future health crisis. Mental health concerns have been reported among older adults [185], but few concrete actions have been taken to mitigate them.

### Strengths and Limitations

Some studies classified older adults as including people below 60 years of age (eg, ≥50 years) [186-188]. Those results were excluded, along with potentially important data, to respect our eligibility criteria and to clearly differentiate the outcomes relative to older versus younger populations. Moreover, some studies focused on older adults with specific conditions, for example, Parkinson disease [36], cancer [39,40] or Alzheimer disease [33], which makes it difficult to differentiate the effects associated with their age from those associated with their condition. These studies were still analyzed, keeping in mind that individuals living with a variety of diagnoses are potentially more vulnerable to encounter negative outcomes related to the secondary effects of COVID-19. Some diagnostic keywords were selected because of the relationship between specific neurological conditions and higher age (eg, dementia and Alzheimer disease). This selection may have resulted in the omission of eligible studies that include older adults with other conditions such as cancer or cardiovascular diseases. However, a limitation in the selected keywords was needed to screen studies within a reasonable timeframe. In addition, the decision was made to exclude studies about COVID-19 survivors, because of the various physical and psychological changes that may be associated with the incidence of this condition. Contradictory results could also be attributable to the variance in health care systems and differences in isolation and protective measures implemented in various countries. Because of constant changing measures across countries, it would have been difficult, if not impossible, to analyze data in such a way. This aspect was, therefore, not considered in our data analysis in order to provide results in a reasonable timeframe. Future studies should take into consideration the country-specific variation in COVID-19 responses. Moreover, it is also possible that the sample age was not mentioned in the abstract or the title of the published papers, which would have resulted in the exclusion of the study during the first stage of screening.

### Implication for Practice and Policy

Results obtained through this rapid review have highlighted the presence of psychological symptoms, decrease in social interactions, exacerbation of ageism, and the deterioration of physical conditions among older adult populations during the COVID-19 pandemic. It is essential that governing bodies and decision makers understand the needs of older adults when making choices regarding the implementation of social distancing measures. They should carefully choose their words when describing this pandemic, to avoid any form of age discrimination in the media.

Older adults represent a heterogeneous group, which could explain the contradictory results found in the sampled literature. Sample demographics should be considered in future studies to identify variables within older adult populations that could be associated with a poorer overall experience with the pandemic, and stronger conclusions could then be made. Indeed, studies that specifically target vulnerable age groups, such as adults living in rural areas [189] and deaf individuals [190], should be conducted to minimize the effects and long-term consequences in such populations. The impact of COVID-19 should be assessed separately according to various living environments in order to identify more at-risk individuals (eg, older adults in the community setting versus long-term care facilities). Future studies should also analyze different protective and risk factors among older adults. For example, it would be interesting to compare the effect of living alone versus living with others, of being in the younger range of the older adult demographic (eg, 60 years old) versus being in the latter range (eg, 85 years old), or of living independently at home versus living in a nursing home. Moreover, the general population could learn from older adults, regarding their resilience, regulation of emotions, and coping strategies, to improve their psychological response during this pandemic. Individual, organizational, and institutional strategies should be established to ensure that older adults are able to maintain social contacts, preserve family ties, and maintain the ability to give or receive help during this pandemic. The effectiveness of various strategies, such as making communication technologies more accessible, providing technology use training, and promoting technological innovations, should also be assessed to enable social interactions despite isolation and protective measures.

## Appendix

Multimedia Appendix 1 Search strategies.

Multimedia Appendix 2 Table S1. Synthesis of the 135 selected studies and their level of evidence.

## Abbreviations

HDM-DCP: Human Development Model–Disability Creation Process
PRISMA: Preferred Reporting Items for Systematic Reviews and Meta-analyses
