# Supplementary material for: Impact of the COVID-19 Pandemic on Older Adults: Rapid Review
Source: JMIR Aging. 2021 Apr 12;4(2):e26474. doi: 10.2196/26474 (PMC8043147; doi:10.2196/26474)
Supplement: Multimedia Appendix 1 [file aging_v4i2e26474_app1.docx]

**Appendix 1. Search Strategies**

| **Databases** | **Search strategies** | | | **Complete search strategies** |
| --- | --- | --- | --- | --- |
|  | **Concepts** | **“Free” vocabulary** | **Controlled vocabulary** |  |
| **MEDLINE VIA PUBMED** | COVID-19 | COVID-19, SARS-CoV-2, coronavirus, "Severe Acute Respiratory Syndrome", pandem*, "severe acute respiratory syndrome coronavirus 2", epidemy, quarantine, epidemic, epidemies, "2019-nCoV" | Severe Acute Respiratory Syndrome | (((COVID-19[Title/Abstract] OR SARS-CoV-2[Title/Abstract] OR coronavirus[Title/Abstract] OR "Severe Acute Respiratory Syndrome"[Title/Abstract] OR pandem*[Title/Abstract] OR "severe acute respiratory syndrome coronavirus 2"[Title/Abstract] OR epidemy[Title/Abstract] OR quarantine[Title/Abstract] OR epidemic[Title/Abstract] OR epidemies[Title/Abstract] OR "2019-nCoV"[Title/Abstract]) OR (Severe Acute Respiratory Syndrome[MeSH:NoExp])) AND ((Psychosocial[Title/Abstract] OR impact*[Title/Abstract] OR determinant*[Title/Abstract] OR outcome*[Title/Abstract] OR effect*[Title/Abstract] OR relation*[Title/Abstract] OR experience*[Title/Abstract] OR psycholog*[Title/Abstract] OR "mental health"[Title/Abstract] OR "emotional well-being"[Title/Abstract] OR anxiety[Title/Abstract] OR "social"[Title/Abstract] OR environment[Title/Abstract] OR "self care"[Title/Abstract] OR "self management support"[Title/Abstract] OR "activities of daily Living"[Title/Abstract] OR "leisure activit*"[Title/Abstract] OR participation[Title/Abstract] OR need*[Title/Abstract]) OR ("Psychosocial support systems"[MeSH:NoExp] OR "psychosocial deprivation"[MeSH:NoExp] OR "self-management"[MeSH:NoExp] OR psychology[MeSH:NoExp] OR "mental health"[MeSH:NoExp] OR "activities of daily Living"[MeSH:NoExp] OR "leisure activities"[MeSH:NoExp]))) AND ((old*[Title/Abstract] OR aged[Title/Abstract] OR 60+[Title/Abstract] OR 65+[Title/Abstract] OR elderly[Title/Abstract] OR senior*[Title/Abstract] OR geriatr*[Title/Abstract] OR aging[Title/Abstract] OR "older adult*"[Title/Abstract] OR "80+"[Title/Abstract] OR dementia[Title/Abstract] OR alzheimer*[Title/Abstract] OR elder[Title/Abstract] OR "geriatric*"[Title/Abstract] OR centenarian*[Title/Abstract]) OR (aged[MeSH:NoExp] OR dementia[MeSH:NoExp] OR "alzheimer disease"[MeSH:NoExp] OR "frail elderly"[MeSH:NoExp] OR "homes for the aged"[MeSH:NoExp])) AND (("2019/01/01"[PDat] : "2020/12/31"[PDat])) |
|  | Impacts | Psychosocial, impact*, determinant*, outcome*, effect*, relation*, experience*, psycholog*, "mental health", "emotional well-being", anxiety, "social", environment, "self care", "self management support", activities of daily Living, leisure activities, participation, need* | Psychosocial support systems, psychosocial deprivation, self-management, psychology, mental health, activities of daily Living, leisure activities |  |
|  | Older adults | old*, aged, 65+, 60+, elderly, senior*, geriatr*, aging, "older adult*", 80+, dementia, alzheimer, elder, geriatric*, centenarian* | Aged, 80 and over, aged, dementia, "alzheimer disease", "frail elderly", "homes for the aged" |  |
| **EMBASE** | COVID-19 | covid-19':ab,ti,kw OR 'sars-cov-2':ab,ti,kw OR 'coronavirus':ab,ti,kw OR 'severe acute respiratory syndrome':ab,ti,kw OR 'pandem*':ab,ti,kw OR 'severe acute respiratory syndrome coronavirus 2':ab,ti,kw OR 'epidemy' OR 'quarantine':ab,ti,kw OR epidemic:ab,ti,kw OR epidemies:ab,ti,kw OR '2019-ncov':ab,ti,kw | covid 19, SARS coronavirus, severe acute respiratory syndrome, pandemic | #11 #3 AND #6 AND #9 AND [embase]/lim AND [1-1-2019]/sd NOT [7-10-2020]/sd  #10 #3 AND #6 AND #9  #9 #7 OR #8  #8 'aged'/de OR 'very elderly'/de OR 'older people'/de OR 'older adult'/de OR 'older adults'/de OR 'geriatrics'/de OR 'elderly care'/de OR 'dementia'/de OR 'alzheimer disease'/de  #7 old*:ab,ti,kw OR aged:ab,ti,kw OR 60+:ab,ti,kw OR 65+:ab,ti,kw OR elderly:ab,ti,kw OR senior*:ab,ti,kw OR geriatr*:ab,ti,kw OR aging:ab,ti,kw OR 'older adult*':ab,ti,kw OR 80+:ab,ti,kw OR dementia:ab,ti,kw OR alzheimer:ab,ti,kw OR elder:ab,ti,kw OR geriatric*:ab,ti,kw OR centenarian*:ab,ti,kw  #6 #4 OR #5  #5 'social psychology'/de OR 'social medecine' OR 'impacts of events scale' OR 'mental health'/de OR 'self care'/de OR 'self management support'/de OR 'social support'/de OR 'daily life activity'/de OR 'leisure'/de  #4 psychosocial:ab,ti,kw OR impact*:ab,ti,kw OR determinant*:ab,ti,kw OR outcome*:ab,ti,kw OR effect*:ab,ti,kw OR relation*:ab,ti,kw OR experience*:ab,ti,kw OR psycholog*:ab,ti,kw OR 'mental health':ab,ti,kw OR 'emotional well-being':ab,ti,kw OR anxiety:ab,ti,kw OR 'self care':ab,ti,kw OR 'social':ab,ti,kw OR environment:ab,ti,kw OR 'self management support':ab,ti,kw OR 'activities of daily living':ab,ti,kw OR 'leisure activit*':ab,ti,kw OR participation:ab,ti,kw OR need*:ab,ti,kw  #3 #1 OR #2  #2 'covid 19'/de OR 'sars coronavirus'/de OR 'severe acute respiratory syndrome'/de OR 'pandemic'/de  #1 'covid 19':ab,ti,kw OR 'sars-cov-2':ab,ti,kw OR 'coronavirus':ab,ti,kw OR 'severe acute respiratory syndrome':ab,ti,kw OR 'pandem*':ab,ti,kw OR 'severe acute respiratory syndrome coronavirus 2':ab,ti,kw OR 'epidemy' OR 'quarantine':ab,ti,kw OR epidemic:ab,ti,kw OR epidemies:ab,ti,kw OR '2019-ncov':ab,ti,kw |
|  | Impacts | psychosocial:ab,ti,kw OR impact*:ab,ti,kw OR determinant*:ab,ti,kw OR outcome*:ab,ti,kw OR effect*:ab,ti,kw OR relation*:ab,ti,kw OR experience*:ab,ti,kw OR psycholog*:ab,ti,kw OR 'mental health':ab,ti,kw OR 'emotional well-being':ab,ti,kw OR anxiety:ab,ti,kw OR 'self care':ab,ti,kw OR 'social':ab,ti,kw OR environment:ab,ti,kw OR 'self management support':ab,ti,kw OR 'activities of daily Living':ab,ti,kw OR 'leisure activit*':ab,ti,kw 0R participation:ab,ti,kw OR need*:ab,ti,kw | social psychology, social medecine, impacts of events scale, mental health, self care, self management support, social support, daily life activity, leisure |  |
|  | Older adults | old*:ab,ti,kw OR aged:ab,ti,kw OR 60+:ab,ti,kw OR 65+:ab,ti,kw OR elderly:ab,ti,kw OR senior*:ab,ti,kw OR geriatr*:ab,ti,kw OR aging:ab,ti,kw OR "older adult*":ab,ti,kw OR 80+:ab,ti,kw OR dementia:ab,ti,kw OR alzheimer:ab,ti,kw OR elder:ab,ti,kw OR geriatric*:ab,ti,kw OR centenarian*:ab,ti,kw | aged/de OR "very elderly"/de OR "older people"/de OR "older adult"/de OR "older adults"/de OR "geriatrics"/de OR "elderly care"/de OR "dementia"/de OR "alzheimer disease"/de |  |
| **PsycINFO and PsycARTICLES via Psycnet** | COVID-19 | COVID-19 or SARS-CoV-2 or coronavirus or "Severe Acute Respiratory Syndrome" or pandem*or "severe acute respiratory syndrome coronavirus 2" or epidemy or quarantine or epidemic or epidemies or "2019-nCoV" | pandemics or epidemics | ((((title: ("old*")))) OR (((title: ("aged")))) OR (((title: ("60+")))) OR (((title: ("65+")))) OR (((title: ("elderly")))) OR (((title: ("senior*")))) OR (((title: ("geriatr*")))) OR (((title: ("aging")))) OR (((title: ("older adult*")))) OR (((title: ("80+")))) OR (((title: ("dementia")))) OR (((title: ("alzheimer")))) OR (((title: ("elder")))) OR (((title: ("geriatric*")))) OR (((title: ("centenarian*")))) OR (((abstract: ("old*")))) OR (((abstract: ("aged")))) OR (((abstract: ("60+")))) OR (((abstract: ("65+")))) OR (((abstract: ("elderly")))) OR (((abstract: ("senior*")))) OR (((abstract: ("geriatr*")))) OR (((abstract: ("aging")))) OR (((abstract: ("older adult*")))) OR (((abstract: ("80+")))) OR (((abstract: ("dementia")))) OR (((abstract: ("alzheimer")))) OR (((abstract: ("elder")))) OR (((abstract: ("geriatric*")))) OR (((abstract: ("centenarian*")))) OR (((MeSH: (Aging)))) OR (((MeSH: (Geriatrics)))) OR (((MeSH: (Gerontology)))) OR (((MeSH: (Dementia)))) OR (((MeSH: ("Alzheimer's Disease")))) OR (((MeSH: ("Older Adulthood"))))) AND ((((((title: (Psychosocial)))) OR (((title: (impact*)))) OR (((title: (anxiety)))) OR (((title: (self care)))) OR (((title: (social)))) OR (((title: (environment)))) OR (((title: (self management support)))) OR (((title: (determinant*)))) OR (((title: (outcome*)))) OR (((title: (effect*)))) OR (((title: (relation*)))) OR (((title: (experience*)))) OR (((title: (psycholog*)))) OR (((title: ("mental health")))) OR (((title: ("emotional well-being")))) OR (((title: ("activit* of daily Li*")))) OR (((title: ("leisure activit*")))) OR (((title: ("participation")))) OR (((title: ("need*")))) OR (((abstract: (Psychosocial)))) OR (((abstract: (impact*)))) OR (((abstract: (anxiety)))) OR (((abstract: (self care)))) OR (((abstract: (social)))) OR (((abstract: (environment)))) OR (((abstract: (self management support)))) OR (((abstract: (determinant*)))) OR (((abstract: (outcome*)))) OR (((abstract: (effect*)))) OR (((abstract: (relation*)))) OR (((abstract: (experience*)))) OR (((abstract: (psycholog*)))) OR (((abstract: ("mental health")))) OR (((abstract: ("emotional well-being")))) OR (((abstract: ("activit*of daily Li*")))) OR (((abstract: ("leisure activit*")))) OR (((abstract: ("participation")))) OR (((abstract: ("need*")))) OR (((MeSH: ("psychosocial development")))) OR (((MeSH: ("psychosocial factors")))) OR (((MeSH: ("mental health")))) OR (((MeSH: ("activities of daily living ")))) OR (((MeSH: ("leisure time")))))) AND ((((((title: (COVID-19)))) OR (((title: (SARS-CoV-2)))) OR (((title: (coronavirus)))) OR (((title: ("Severe Acute Respiratory Syndrome")))) OR (((title: (pandem*)))) OR (((title: ("severe acute respiratory syndrome coronavirus 2")))) OR (((title: (epidemy)))) OR (((title: (quarantine)))) OR (((title: (epidemic)))) OR (((title: (epidemies)))) OR (((title: ("2019-nCoV"))))) OR (((abstract: (COVID-19)))) OR (((abstract: (SARS-CoV-2)))) OR (((abstract: (coronavirus)))) OR (((abstract: ("Severe Acute Respiratory Syndrome")))) OR (((abstract: (pandem*)))) OR (((title: ("severe acute respiratory syndrome coronavirus 2")))) OR (((abstract: (epidemy)))) OR (((abstract: (quarantine)))) OR (((abstract: (epidemic)))) OR (((abstract: (epidemies)))) OR (((abstract: ("2019-nCoV")))) OR (((MeSH: (pandemics)))) OR (((MeSH: (epidemics))))))) AND Year: 2019 To 2020 |
|  | Impacts | Psychosocial or impact* or determinant* or outcome* or effect* or relation* or experience* or psycholog* or "mental health" or "emotional well-being" or anxiety or self care or social or environment or self management support or activities of daily Living or leisure activities or participation or need | “psychosocial development” or “psychosocial assessment” or “psychosocial factors” or “mental health” or activities of daily living or leisure time |  |
|  | Older adults | old*, aged, 60+, 65+, elderly, senior*, geriatr*, aging, "older adult*", 80+, dementia, alzheimer, elder, geriatric*, centenarian* | Aging OR Geriatrics OR Gerontology OR Dementia OR "Alzheimer's Disease" OR "Older Adulthood" |  |
| **CINAHL VIA EBSCOHOST** | COVID-19 | COVID-19 or SARS-CoV-2 or coronavirus or "Severe Acute Respiratory Syndrome" or pandem*or "severe acute respiratory syndrome coronavirus 2" or epidemy or quarantine or epidemic or epidemies or "2019-nCoV" | (MM "SARS Virus") OR (MM "Coronavirus") | TI ( COVID-19 or SARS-CoV-2 or coronavirus or "Severe Acute Respiratory Syndrome" or pandem*or "severe acute respiratory syndrome coronavirus 2" or epidemy or quarantine or epidemic or epidemies or "2019-nCoV" ) OR AB ( COVID-19 or SARS-CoV-2 or coronavirus or "Severe Acute Respiratory Syndrome" or pandem*or "severe acute respiratory syndrome coronavirus 2" or epidemy or quarantine or epidemic or epidemies or "2019-nCoV" ) OR ( (MM "SARS Virus") OR (MM "Coronavirus") ) AND TI ( Psychosocial or impact* or determinant* or outcome* or effect* or relation* or experience* or psycholog* or "mental health" or "emotional well-being" or anxiety or "self care" or social or environment or "self management support" or "activities of daily Living" or "leisure activit*" or participation or need* ) OR AB ( Psychosocial or impact* or determinant* or outcome* or effect* or relation* or experience* or psycholog* or "mental health" or "emotional well-being" or anxiety or "self care" or social or environment or "self management support" or "activities of daily Living" or "leisure activit*" or participation or need* ) OR ( (MM "Psychosocial Aspects of Illness") OR (MM "Diagnosis, Psychosocial") OR (MM "Support, Psychosocial") or (MM "activities of daily living") OR (MM "leisure activities") ) AND TI ( old* or aged or "60+" or "65+" or elderly or senior* or geriatr* or aging or "older adult*" or "80+" or dementia or alzheimer or elder or geriatric* or centenarian* ) OR AB ( old* or aged or "60+" or "65+" or elderly or senior* or geriatr* or aging or "older adult*" or "80+" or dementia or alzheimer or elder or geriatric* or centenarian* ) OR ( (MM "Aged") OR (MM "Aged, 80 and Over") OR (MM "Dementia") OR (MM " Alzheimer's Disease") OR (MM "Elder Abuse") OR (MM "Frail Elderly") ) |
|  | Impacts | Psychosocial or impact* or determinant* or outcome* or effect* or relation* or experience* or psycholog* or "mental health" or "emotional well-being" or anxiety or self care or social or environment or self management support or activities of daily Living or leisure activities or participation or need* | (MM "Psychosocial Aspects of Illness") OR (MM "Diagnosis, Psychosocial") OR (MM "Support, Psychosocial") or activities of daily living or leisure activities |  |
|  | Older adults | old* or aged or or “60+” or "65+" or elderly or senior* or geriatr* or aging or "older adult*" or "80+" or dementia or alzheimer or elder or geriatric* or centenarian* | (MM "Aged") OR (MM "Aged, 80 and Over") OR (MM "Dementia") OR (MM " Alzheimer's Disease") OR (MM "Elder Abuse") OR (MM "Frail Elderly") |  |
| **AGELINE VIA EBSCOHOST** | COVID-19 | COVID-19, SARS-CoV-2, coronavirus, "Severe Acute Respiratory Syndrome", pandem*, "severe acute respiratory syndrome coronavirus 2", epidemy, quarantine, epidemic, epidemies, "2019-nCoV" | N/A | TI ( COVID-19 or SARS-CoV-2 or coronavirus or "Severe Acute Respiratory Syndrome" or pandem*or "severe acute respiratory syndrome coronavirus 2" or epidemy or quarantine or epidemic or epidemies or "2019-nCoV" ) OR AB ( COVID-19 or SARS-CoV-2 or coronavirus or "Severe Acute Respiratory Syndrome" or pandem*or "severe acute respiratory syndrome coronavirus 2" or epidemy or quarantine or epidemic or epidemies or "2019-nCoV" ) AND TI ( Psychosocial or impact* or determinant* or outcome* or effect* or relation* or experience* or psycholog* or "mental health" or "emotional well-being" or anxiety or "self care" or social or environment or "self management support" or "activities of daily Living" or "leisure activit*" or participation or need* ) OR AB ( Psychosocial or impact* or determinant* or outcome* or effect* or relation* or experience* or psycholog* or "mental health" or "emotional well-being" or anxiety or "self care" or social or environment or "self management support" or "activities of daily Living" or "leisure activit*" or participation or need* ) OR (MM "Psychosocial needs") OR (MM "social needs") OR (MM "emotional needs") OR (MM "mental health") OR (MM "psychological well being) AND TI ( old* or aged or or "60+" or "65+" or elderly or senior* or geriatr* or aging or "older adult*" or "80+" or dementia or alzheimer or elder or geriatric* or centenarian* ) OR AB ( old* or aged or "60+" or "65+" or elderly or senior* or geriatr* or aging or "older adult*" or "80+" or dementia or alzheimer or elder or geriatric* or centenarian* ) OR (MM "older adults") OR (MM "85+") OR (MM "65+") OR (MM "old old") OR (MM "centenarians") OR (MM "aging") OR (MM "frail elderly") OR (MM "dementia") OR (MM "alzheimers disease") |
|  | Impacts | Psychosocial or impact* determinant* or outcome* or effect* or relation* or experience* or psycholog* or "mental health" or "emotional well-being" or anxiety or self care or social or environment or self management support or activities of daily Living or leisure activities or participation or need* | Psychosocial needs, social needs, emotional needs, mental health, psychological well being, activities of daily living, leisure activities |  |
|  | Older adults | old* or aged or or “60+” or "65+" or elderly or senior* or geriatr* or aging or "older adult*" or "80+" or dementia or alzheimer or elder or geriatric* or centenarian* | (MM "older adults") OR (MM "85+") OR (MM "65+") OR (MM "old old") OR (MM "centenarians") OR (MM "aging") OR (MM "frail elderly") OR (MM "dementia") OR (MM "alzheimers disease") |  |
