# Supplementary material for: Impact of the COVID-19 Pandemic on Older Adults: Rapid Review
Source: JMIR Aging. 2021 Apr 12;4(2):e26474. doi: 10.2196/26474 (PMC8043147; doi:10.2196/26474)
Supplement: Multimedia Appendix 2 [file aging_v4i2e26474_app2.docx]

Table S1. Synthesis of the 135 selected studies and their level of evidence.

| Author (year) | Country | Title | Study design | Objective | Population | Outcomes and results | Level of evidence |
| --- | --- | --- | --- | --- | --- | --- | --- |
| Al-Hashel et al. (2020)^45^ | Kuwait | Impact of coronavirus disease 2019 (COVID-19) pandemic on patients with migraine: a web-based survey study | Cross-sectional study | Investigate the impact of COVID-19 on migraine frequency and severity, medical care, doctor-patient communication, in addition to overall psychosocial health and specific COVID-19 concerns in Kuwait. | 1018 patients with a medical diagnosis of migraine (>60 years old: 12 (1.2%)) | In patients of more than 60 years old, 6 out of 12 had an increase in migraine days and 7/12 had an increase in migraine severity. There was no association with those factors and with patients’ age. | 4 |
| Al Sulais et al. (2020)^159^ | Saudi Arabia | The psychological impact of COVID-19 pandemic on physicians in Saudi Arabia: A cross-sectional study | Cross-sectional study | Determine the psychological impact of the COVID-19 pandemic on physicians in Saudi Arabia and assess the major concerns and possible risk factors that may contribute to such an impact. | 529 physicians practicing various specialties of medicine (60 and older: 12 (2.30%)) | Physicians older than age 60 were less likely to experience the feeling of being isolated. | 4 |
| Antunes et al. (2020)^87^ | Portugal | Exploring lifestyle habits, physical activity, anxiety and basic psychological needs in a sample of Portuguese adults during COVID-19 | Cross-sectional study | To better understand the lifestyle habits of the Portuguese adult population during the COVID-19 pandemic, namely regarding sleeping habits, eating habits and physical activity habits; and to explore the anxiety levels and the satisfaction of basic psychological needs during the COVID-19 pandemic in Portuguese adults. | 1404 Portuguese  respondents (36.4 ± 11.7 years of age), ranging from 18 to 89 years of age; 19 had more than 65 years old. | In people with more than 65 years old: 68.4% had their usual sleep duration, 78.9% were satisfied with their sleep quality, 31.6% ate more often, 21.1% ate more and 47.4% had a careful food selection. 36.6% spent more than 3 hours per day watching, reading or listening about the COVID-19. They had a total energy expenditure (METS) of 2448 ± 3435, an anxiety state of 44.6 ± 9.2 and an anxiety trait of 34.5 ± 6.8. Changes were more present in the group 18-34. | 4 |
| Arpino et al. (2020)^88^ | France, Italy, and Spain | Older People's Non-Physical Contacts and Depression During the COVID-19 Lockdown | Cross-sectional study | Examine to what extent intergenerational and other types of non-physical contacts have reduced the risk of increased perceived depressive feelings during the lockdown for people aged 50+. | 4,207 individuals of 50 years old and more | Among individuals aged 70+, those who did not change their non-intergenerational contacts during the lockdown reduced their probability of increased perceived depressive feelings of about 10 percentage points compared with those who decreased the frequency of such contacts. | 4 |
| Bailey et al. (2020)^43^ | United States | Changes in COVID-19 Knowledge, Beliefs, Behaviors, and Preparedness Among High-Risk Adults from the Onset to the Acceleration Phase of the US Outbreak | Longitudinal two-wave telephone survey | To assess how high-risk adults’ COVID-19 knowledge, beliefs, behaviors, and sense of preparedness changed from the onset of the US outbreak (March 13–20, 2020) to the acceleration phase (March 27–April 7, 2020). | 588 predominately older adults with ≥ 1 chronic condition  (60-69: 36.6%, ≥70: 27.4%) | Changed daily routine was reported by 64.6% of adults of ≥70 and 75.4% of adults of 60-69 years old. Changed plans were reported by 83.6% of adults of ≥70 and 87.4% of adults of 60-69 years old. | 2b |
| Barber et al. (2020)^48^ | United States | COVID-19 Worries and Behavior Changes in Older and Younger Men and Women | Cross-sectional study | Whether older adults–and particularly older men—would report the least amount of COVID-19 worry and also fewer COVID-19 behavior changes. | Convenience sample of community-dwelling U.S. residents, 146 were younger (18–35 years) and 156 were older in age (65–81 years) | Older adults perceived the risks of COVID-19 to be higher than did younger adults. Older men were less worried about COVID-19 than their younger counterparts. Compared with the other participants, older men had also implemented the fewest behavior changes. | 4 |
| Barros et al. (2020)^147^ | Brazil | Report on sadness/depression, nervousness/anxiety and sleep problems in the Brazilian adult population during the COVID-19 pandemic | Cross-sectional study | Analyze the frequency of sadness, nervousness, and sleep disorders during the COVID-19 pandemic in Brazil, identifying the most affected demographic segments. | 45,161 respondents, 20.3% were aged 60 or over | Whereas 27.5% of elderly people reported feeling sad or depressed often or all the time, this feeling affected 53.8% of young adults; 9.1% of young adults reported feeling sad all the time, this percentage being 2.9 times greater than among elderly people. Feeling nervous or anxious all the time or nearly all the time was also reported by 31.7% of elderly people and by 69.5% of young adults. Prevalence of feeling anxious/nervous all the time among young adults was 3.7 times greater than among elderly people. For young adults, worsening of prior sleep problems was two times greater than the elderly, and the onset of sleep problems was 84% greater. | 4 |
| Bäuerle et al. (2020)^89^ | Germany | Mental Health Burden of the COVID-19 Outbreak in Germany: Predictors of Mental Health Impairment | Cross-sectional study | Assess individual changes in mental health and health status before and after the COVID-19 outbreak, and to explore potential predictors of change. | 15 037 respondents (65 to 74 years: 662 (4.4%), ≥75 years old: 109 (0.8%)) | Increased in psychological symptoms were noted after the COVID-19 outbreak compared with before: major depression symptoms (64+, before: 3.5%; after: 8.7%), severe generalized anxiety symptoms (64+, before: 4.1%; after: 10.0%), elevated distress (64+, before: 25.9%; after: 43.7%). 64+ were the group age with the lowest scores. | 4 |
| Bergman et al. (2020)^143^ | Israel | COVID-19 health worries and anxiety symptoms among older adults: The moderating role of ageism | Cross-sectional study | Examining the role of ageism in the connection between COVID-19 health worries and anxiety symptoms among older adults and explored the moderating role of ageism as strengthening this connection. | 243 older adults (age range 60–92; M = 69.75, SD = 6.69) | Health worries and ageism were positively associated with anxiety symptoms. The connection between health worries and anxiety symptoms was more pronounced among older adults with high ageism levels. | 4 |
| Bobes-Bascarán  et al. (2020)^90^ | Spain | Early Psychological Correlates Associated With COVID-19 in A Spanish Older Adult Sample | Cross-sectional study | Examine the early psychological correlates associated with the COVID-19 pandemic and lockdown on the mental health of a Spanish older adult sample, and to analyze the influence of past mental disorder (PMD) and current mental disorder (CMD) on those correlates. | 2,194 individuals aged 60 years or more, mean age: 65.62 (5.05) | 25.6% of the sample could be considered as having symptoms of depression, 3.6% of anxiety, and 11% of stress. | 4 |
| Boschuetz et al. (2020)^71^ | United States | Changes in Alcohol Use Patterns in the United States During COVID-19 Pandemic | Cross-sectional survey | Assess changes in alcohol use patterns as a result of social distancing measures. | 407 participants including 33 (8%) aged > 65 years old | Authors did not observe important change in Alcohol Use Disorders Identification Test (AUDIT-C) scores during social distancing vs. pre-social distancing in participants aged 65 years and older (+0.21 points). Age was not a significant factor in score change (p=0.35). | 4 |
| Boutoleau-Bretonnière et al. (2020)^34^ | France | The Effects of Confinement on Neuropsychiatric Symptoms in Alzheimer's Disease During the COVID-19 Crisis | Cross-sectional study | Investigate whether the occurrence and severity of neuropsychiatric symptoms increased during the confinement. | 38 participants (23 women; M age = 71.89 years, SD = 8.24) with a clinical diagnosis of probable AD and the patients’ caregivers (M age = 68.43 years, SD = 12.16) | 10 demonstrated neuropsychiatric changes during the confinement. Among them, the duration of confinement was significantly correlated with the severity of symptoms as well as with their caregivers’ distress. | 4 |
| Brooke et al. (2020)^91^ | England, Republic of Ireland | Older people's early experience of household isolation and social distancing during COVID-19 | Longitudinal qualitative study | Explore changes to daily life experienced by the over-70s during the first two weeks of household isolation, social distancing and shielding, and their initial perceptions and plans to support them through the COVID-19 pandemic. | 19 participants aged 70 or older, mean age: 77.00 (5.77) | Within the first two weeks of enforced social distancing measures, all participants discussed their plans for the immediate future, including having enough tasks to do within their house and garden to keep them busy, which would support them through the pandemic and provide them with structure to their days. All participants discussed feeling blessed, lucky and fortunate, for example having the ability to use social media to stay in contact with their friends and family members. Due to their age, participants discussed the possibility of death if they came into contact with the virus, while simultaneously discussing how they were appreciative of having had a long life, and not being afraid of death, but still had some life left to live. | 2b |
| Bruine de Bruin (2020)^49^ | United States | Age differences in COVID-19 risk perceptions and mental health: Evidence from a national US survey conducted in March 2020 | Cross-sectional study | Examine whether older adult age was associated with lower risk perceptions for COVID-19 and with less depression and anxiety. | 6,666 adults, aged 18–100 (M = 48.56, SD = 16.62)  For all risks except job loss: 60–69 = 1,199, ≥70 = 816; For job loss: N = 509 for age group 60–69, N = 131 for age group ≥70; Depression and anxiety: N = 1,199 for age group 60–69, N = 816 for age group ≥70. | Risk perceptions of: Getting COVID-19 (60-69: 18%; 70: 16%), Dying if getting COVID-19 (60-69: 18%; ³70: 25%), getting quarantined (60-69: 18%; 70: 16%), losing job (60-69: 11%; 70: 16%), running out of money (60-69: 10%; 70: 5%); Presence of: Depression and anxiety (60-69: 7%; 70: 4%), depression (60-69: 9%; 70: 5%), anxiety (60-69: 11%; 70: 7%) | 4 |
| Bu et al. (2020)^92^ | United Kingdom | Who is lonely in lockdown? Cross-cohort analyses of predictors of loneliness before and during the COVID-19 pandemic | Cross-sectional study with two different time points | Compare sociodemographic predictors of loneliness before and during the COVID-19 pandemic. | Adults captured before the pandemic n=31,064; 60+= 34.7%) and during the pandemic (n=60,341; 60+=29.9%) | Compared with adults aged 60+, adults aged 18-30 years were more likely to be lonely, before the pandemic (coef = 1.01, 95% CI: 0.89-1.12), and during the pandemic (coef = 1.58, 95% CI: 1.48-1.68). | 4 |
| Burhamah  et al. (2020)^158^ | Kuwait | The psychological burden of the COVID-19 pandemic and associated lockdown measures: Experience from 4000 participants | Cross-sectional study | Assess the impact of COVID19 outbreak on mental health in Kuwait, and to explore the potential influencing risk factors. | 4,132 respondents, including 363 (8.8%) aged 61-70 and 39 (0.9%) aged ≥ 71 | Those between the age of 61 and 70 displayed a low tendency for depression and anxiety, with younger groups having a higher tendency. | 4 |
| Cagnin et al. (2020)^93^ | Italy | Behavioral and psychological effects of coronavirus disease-19 quarantine in patients with dementia | Cross-sectional study | Assess modifications of neuropsychiatric symptoms during quarantine in patients with dementia and their caregivers | 4,913 family caregivers of patients with diagnosis of Alzheimer’s disease (AD), dementia with Lewy bodies (DLB), frontotemporal dementia (FTD), and vascular dementia (VD). Patients had a mean age of 78.3 ± 8.2. | Increased behavioral and psychological symptoms were reported in 59.6% of patients as worsening of pre-existing symptoms (51.9%) or as new onset (26%) and requested drug modifications in 27.6% of these cases. Irritability, apathy, agitation, and anxiety were the most frequently reported worsening symptoms, and sleep disorder and irritability the most frequent new symptoms. Worsening of sleep disorder and hallucinations were more frequent in DLB, while worsening of wandering and change of appetite were more frequently reported in FTD. Anxiety was more frequently reported in AD and DLB. On the contrary, some symptoms increased similarly across disease groups such as apathy. Having AD was associated with an increased risk of anxiety, DLB with hallucinations and sleep disorder, and FTD with wandering and change of appetite. On the opposite, AD and FTD had lower risk of worsening hallucinations and FTD and VD to develop worsening of anxiety. No significant associations were found between type of dementia and type of new BPSD. | 4 |
| Callow et al. (2020)^50^ | United States and Canada | The mental health benefits of physical activity in older adults survive the covid-19 pandemic | Descriptive cross-sectional study | Determine the relationship between the amount and intensity of physical activity performed by older adults in North America and their depression and anxiety symptoms while currently under social distancing guidelines (SDG) for the COVID-19 pandemic. | 1,046 older adults (ages≥50); 60−69 (424; 0.5%), 70−79 (257; 24.5%), 80−89 (67; 6.4%), >90 (5;0.4%) | Compared to individuals who were 50−59 years old, individuals aged 70−79 and 80−89 years old were predicted to have depression scores 2.07 and 3.32 points lower, respectively, after controlling for total physical activity, sex, and education. | 4 |
| Callow et al. (2020)^51^ | United States | Older Adults’ Intention to Socially Isolate Once COVID-19 Stay-at-Home Orders Are Replaced With “Safer-at-Home” Public Health Advisories: A Survey of Respondents in Maryland | Descriptive cross-sectional study | Examine the opinion and behaviors of older adults regarding Coronavirus Disease 2019 (COVID-19), social distancing practices, stay-at-home orders, and hypothetical public policy messaging strategies. | Sample size of 242 adults of 60 years old or more | 24.4% were minimally anxious, 56.2% were mildly anxious, 10.7% were moderately anxious, and 8.9% would be considered severely anxious. | 4 |
| Canet-Juric et al. (2020)^162^ | Argentina | A Longitudinal Study on the Emotional Impact Cause by the COVID-19 Pandemic Quarantine on General Population | Longitudinal study | Analyze the longitudinal emotional effect of the social, preventive and mandatory isolation established due to the epidemiological COVID-19 situation in Argentina. | 6057 participants, including 415 (6.9%) aged 60 years or older | For the 60 years or older, the mean (SD) depressive symptoms were 5.50 (5.41) at the beginning of the lockdown (T1) and 5.86 (5.82) fourteen days after (T2) (p=0.32). Anxiety level was 0.98 (0.50) at T1 and 0.92 (0.45) at T2 (p=0.43). Negative affect was 15.31 (4.73) at T1 and 14.83 (4.92) at T2 (p=0.28. Positive affect was 28.15 (7.15) at T1 and 27.72 (7.15) at T2 (p=0.004). | 2b |
| Capozzo et al. (2020)^37^ | Italy | Telemedicine for Delivery of Care in Frontotemporal Lobar Degeneration During COVID-19 Pandemic: Results from Southern Italy | Cross-sectional study | Evaluate multidisciplinary assessment of patients with FTD using telehealth during the COVID-19 pandemic | Sample size of 32 FTD patients (28 done with caregivers and 4 with caregivers and patients), aged 66.25 ± 9.76 | Significant worsening since previous clinical assessment was mostly in behavior (56%), language (47%), and cognitive functions (53%). Memory was described as worsened in 17 out 32 patients. Significant sleep disturbances were reported in 25% of patients since the start of quarantine time. | 4 |
| Carriedo et al. (2020)^94^ | Spain | COVID-19, Psychological Well-being and Physical Activity Levels in Older Adults During the Nationwide Lockdown in Spain | Cross-sectional study | Examine the psychological well-being of older adults during the home isolation due to the COVID-19 pandemic and to investigate whether meeting the World Health Organization’s global recommendations on physical activity (PA) for health is associated with their resilience, affect, and depressive symptoms. | 483 citizens whose ages ranged from 60 to 92 years, *M* = 65.49 (5.14) | Older adults who regularly engaged in vigorous (VPA) and moderate-vigorous physical activity (MVPA) during the quarantine reported higher scores in resilience (Locus, Self-efficacy, and Optimism), positive affect, and lower in depressive symptoms. | 4 |
| Carson et al. (2020)^95^ | England | Winter is coming: age and early psychological concomitants of the Covid-19 pandemic in England | Cross-sectional quantitative study | Demonstrate early psychological concomitants of the Covid-19 pandemic in England on a sample of younger and older people. | 495 participants: younger participants (18 to 25 years, n = 391) and older participants (60 to 80 years, n = 104) | Flourishing levels (positive emotions, engagement, relationships, meaning and accomplishment) for older participants were significantly higher (M = 107.96) than for younger participants (M = 97.80). Younger participants scored significantly higher on the ONS4 for anxiety and lower than the older participants for happiness, life satisfaction and having a worthwhile life. Levels of psychological distress (CORE-10) were also significantly lower for older participants (M = 9.06) than for younger participants (M = 14.61). Finally, younger participants scored significantly higher on the Brief UCLA Loneliness Scale (M = 6.05) than older participants (M = 4.64). | 4 |
| Chen et al. (2020)^52^ | United States | Reactions to COVID-19, information and technology use, and social connectedness among older adults with pre-frailty and frailty | Cross-sectional study | Report on: (1) the impact of the pandemic on daily life; (2) preparedness, perceptions, and behavior; (3) information and technology use; and (4) social impacts on older adults. | 10 participants, mean age: 75.3±6.25, range: 66–84 | Most participants expressed negative emotional reactions, including stress, anxiety, and worry, towards COVID-19. Participants reported engaging in preventive behaviors, including social distancing. They often considered difficult changes to their daily routines. Participants reported stopping going to gyms and group exercises. The content that participants contributed confirmed that most participants felt isolated due to pandemic circumstances. | 4 |
| Ciancio et al. (2020)^53^ | United States | Know your epidemic, know your response: Early perceptions of COVID-19 and self-reported social distancing in the United States | Cross-sectional study | Document individuals’ perceptions of the risks and consequences of the COVID-19 pandemic in the United States. Show the average levels of risk perceptions of getting infected with Covid-19, the mortality and economic risks associated with the pandemic as well as the variation in these risk perceptions by basic demographic characteristics. | 5,414 respondents aged 18 years and older,  mean age= 48.1±17.2 | Compared with participants 59 or younger, participants aged 60 y or older perceived they had lower chances of getting infected, more chances of dying if infected, lower chances of running out of money, lower chances of losing jobs (assessed only among those that had a job) and were slightly less likely to engage in physical distancing. Younger U.S. residents were at least as likely to have taken social distancing measures as older persons. | 4 |
| Clotworthy et al. (2020)^96^ | Denmark | 'Standing together - at a distance': Documenting changes in mental-health indicators in Denmark during the COVID-19 pandemic | Longitudinal study | Investigate how the pandemic and its related public-health measures affected people’s worries, quality of life, social isolation, relationships and everyday behaviour. | 3 groups: general population, families with children and older people. Older people (n=1059), mean age: 72, range: 65-89 | Older people were more worried about becoming seriously ill themselves and not being able to see their family and friends. Their level of social isolation increased throughout most of the observation period but began to decrease around 13 April. Similarly, older people’s quality of life decreased during the first weeks of the lockdown but then increased from around 9 April. Compared with other groups, older people had generally higher levels of worry throughout the observation period, experiencing the greatest decrease in quality of life and the greatest increase in social isolation. | 2b |
| Cohen et al. (2020)^160^ | Argentina | COVID-19 Epidemic in Argentina: Worsening of Behavioral Symptoms in Elderly Subjects with Dementia Living in the Community | Cross-sectional study | Study to what extent mandatory quarantine imposed due to COVID-19 had affected behavioral symptoms in subjects with dementia after the first 8 weeks of quarantine. | Family caregivers (n = 119) of persons with AD or related dementia living at home. Mean age of the family members was 58.61±13.60 years, 26.3% (n=30) were aged 65-85, and 2.6% (n=3) were older than 85 years of age. Older adults with dementia (n=119): mean age=81.16±7.03, 71.4% (n=85) of the subjects were aged 65-85, and 26.9% (n=32) had more than 85 years of age. | Important findings were increased anxiety (43% of the sample), insomnia (28% of the subjects), depression (29%), worsening gait disturbance (41%), and increase use of psychotropics to control behavioral symptoms. When we compared the frequency of behavioral symptoms within each dementia group category, we found that anxiety, depression, and insomnia were more prevalent in subjects with mild dementia compared to subjects with severe dementia. Rehabilitation services had been discontinued in most subjects due to the quarantine. | 4 |
| Constant et al. (2020)^131^ | France | Socio-Cognitive Factors Associated with Lifestyle Changes in Response to the COVID-19 Epidemic in the General Population: Results from a Cross-Sectional Study in France | Cross-sectional study | Assess changes in lifestyles in the general population in response to coronavirus disease 2019 (COVID-19) lockdown and the influence of COVID-19 perceptions, as assessed by the Extended Parallel Process Model (EPPM), on these changes. | 4005 individuals, n=1032 (25.8%) were 60 and older | Estimate of unhealthy changes decreased with age older than 60 years. Estimates of higher drinking decreased with age (60 years or older: OR = 0.56 [0.34–0.91], 40–59 years: OR = 0.61 [0.43–0.87]; reference: 18–39 years). | 4 |
| Dai et al. (2020)^157^ | Malaysia | Perception of health conditions and test availability as predictors of adults’ mental health during the covid-19 pandemic: A survey study of adults in Malaysia | Cross-sectional study | Examine individuals’ perception of health conditions and test availability as potential predictors of mental health—insomnia, anxiety, depression, and distress—during the COVID-19 pandemic. | 669 adults (60-71 years old: 32 (4.78%)) | Younger adults reported worse mental health (age was found to be a predictor of mental health problems for the general population in Malaysia). Age negatively predicted insomnia, anxiety, depression, and distress. | 4 |
| Daly et al. (2020)^54^ | United States | Depression reported by US adults in 2017-2018 and March and April 2020 | Cross sectional study and longitudinal study | Assess depression levels before and during the COVID-19 pandemic in the United States. | Pre-pandemic: N = 5,075 adults; During pandemic: March N = 6,819 adults and April N = 5,428 adults | Statistically significant increases in the probability of depression from 2017 to 2018 to April 2020 were identified for all population subgroups examined with the exception of those aged 65 years and over. Those aged 65+ reported low levels of depression during the pandemic. | 4 and 2b |
| de Maio Nascimento  (2020)^148^ | Brazil | Covid-19: U3A students' report on the impacts of social isolation on physical and mental health and access to information about the virus during the pandemic | Descriptive cross-sectional study | Understand the following research questions: (1) Is social isolation impacting the physical and mental health of older adults? And what are its consequences? (2) What strategies are being used by older adults to keep themselves informed about Covid-19? | 35 older adults (60–81 years old), with a mean age of 70.5 ± 4.7 years | The interviewees underlined the feeling of sadness that the news produces. There were manifestations of anxiety among older adults, mainly because they belong to the risk group. The testimonies indicated that compliance with the hygiene recommendations to avoid contagion from COVID-19 is a psychological burden on the daily lives of older adults. The majority reported feeling low vitality. The reports clearly showed changes in levels of strength, aerobic resistance, agility, and flexibility, with consequences on functionality. They also highlighted changes in their eating habits, besides the loss of muscle mass. | 4 |
| Di Sebastiano et al. (2020)^55^ | Canada | Don't Walk So Close to Me: Physical Distancing and Adult Physical Activity in Canada | Quasi-experimental study | Determine device-measured physical activity levels immediately prior to and following the implementation of physical distancing measures in Canada to provide evidence for the development of physical activity recommendations for future pandemics or second wave infections. | Whole cohort: N=23,173 (65+ years: 1,680 (7.3%)); Complete date cohort: N=2,338 (65+ years: 186 (8.0%)) | Older adults (55–64 and 65+ year old) recorded less light physical activity and steps compared to younger adults (25–34, 35–44, and 45–55-year-olds). These findings persisted over the 10 weeks of data collection, which suggests that older adults recorded less incidental physical activity prior to the pandemic which continued into the pandemic. | 2b |
| Doshi et al. (2020)^155^ | India | Assessing Coronavirus Fear in Indian Population Using the Fear of COVID-19 Scale | Cross-sectional study | Determinate the level of fear of COVID-19 among Indian residents using the Fear of COVID-19 Scale (FCV-19S) and compare it with demographic variables. | 1499 respondents:  47 (3.1%) respondents had more than 60 years | On 5-level Likert scale, compared with 20-40 y old, participants 60 y and older were more likely to disagree to the statement "You cannot sleep because you are worrying about getting coronavirus disease-19" (mean score 1.98 ± 0.95 vs. 1.83 ± 0.89, respectively); and to the statement "Your heart races or palpitates when you think about coronavirus disease-19" (mean score 2.10 ± 1.02 vs. 1.79 ± 0.88, respectively). | 4 |
| El Haj et al. (2020)^33^ | France | High depression and anxiety in people with Alzheimer's disease living in retirement homes during the covid-19 crisis | Cross-sectional study | Evaluate symptoms of depression and anxiety in participants with AD who live in retirement homes in France during the Covid-19 crisis. | Fifty-eight participants with a clinical diagnosis of probable AD (37 women and 21 men; M age = 71.79 years, SD = 5.54; M years of formal education = 9.26, SD = 2.33) | Participants reported significant higher depression during than before the Covid-19 crisis. Participants also reported higher anxiety during than before the Covid-19 crisis. | 4 |
| Emerson (2020)^56^ | United States | Coping with being cooped up: Social distancing during COVID-19 among 60+ in the United States | Cross-sectional study | Explore how adults aged 60 and older in the US are coping with social distancing and sheltering in place during the COVID-19 pandemic. | 833 respondents living in the US, aged 60 to 85 years old, with 62.8% in the younger (aged 60-70) category. | 42.9% of respondents reported feeling lonely some or most days. People reported being stressed (36.9%), and/or being lonelier (30.9%). Nearly 1/3 stated that their sense of loneliness increased during the time of social distancing. 27% slept more than usual and 15.8% reporting less sleep. Also, 37% engaged in less physical activity, 56% engaged in more solitary activities than usual and 48% engaged in fewer in person activities than usual. 66% used more social media and 60% send/received more text messages than usual. Alcohol intake increased for 12.2% of the sample. Since social distancing, over one-third (35.4%) of respondents’ report eating more than usual, and 11.1% ate less than usual. | 4 |
| Ettman et al. (2020)^57^ | United States | Prevalence of depression symptoms in US adults before and during the COVID-19 pandemic | Cross-sectional study with two different time points | Estimate the prevalence of and risk factors associated with depression symptoms among US adults during vs before the COVID-19 pandemic. | Before COVID: 5065 (≥60 years old: 1819 (28%); during COVID-10: 1441 participants (≥60 years old: 360 (29.7%) | Depression symptoms: Increase during the COVID-19 for people aged ≥60 (50 (14.9%)) compared with before COVID-19 (165 (7.9%)). However, higher scores were found in younger groups. | 4 |
| Fernández et al. (2020)^161^ | Argentine | Psychological distress associated with COVID-19 quarantine: Latent profile analysis, outcome prediction and mediation analysis | Cross-sectional study | Characterize the subtypes of psychological distress associated with quarantine, assess its prevalence, explore risk/protective factors, and possible mechanisms. | 4408 Argentine volunteers ranging from 18 to 92 years (>65 years old: 777 (17.6%)) | Older adults (>65 years) were associated with lesser odds of intense psychological distress. Being an older adult was associated with better mental health outcomes. | 4 |
| Ferrante et al. (2020)^132^ | Italy | Did social isolation during the SARS-CoV-2 epidemic have an impact on the lifestyles of citizens? | Cross-sectional study | Investigate and ana- lyse the impact of COVID-19 lockdown on daily habits through a lifestyle survey, with a particular focus on PA, nutrition, alcohol intake, and smoking, taking into account potential differences related to sociodemographic characteristics. | 7 847 participants including 446 (5.7%) participants of 70 years or older | Among participants of 70 years or older, during the COVID-19 social isolation period, 56.5% (p=0.50) reduced physical activity, 4.8% (p=0.006) increased alcohol consumption and 36% (p=0.47) increased cigarette smoking. 21.7% (p=0.39) reported increase in unhealthy food and 26.6% (p=0.28) increase in healthy food. | 4 |
| Findlay et al. (2020)^64^ | Canada | Understanding the Perceived Mental Health of Canadians During the COVID-19 Pandemic | Cross-sectional study | Report on the self-perceived mental health of Canadians during the COVID-19 pandemic. | 4,627 of persons of 15 years of age or older living, 21% were 65 years old or older | 26.8 % had excellent/very good perceived mental health and 12.7% had good/fair/poor perceived mental health. | 4 |
| Fong et al. (2020)^74^ | China | Relationships between Physical and Social Behavioural Changes and the Mental Status of Homebound Residents in Hong Kong during the COVID-19 Pandemic | Cross-sectional study | Examine the changes in physical health, mental health, and social well-being experienced by local residents who were homebound during the pandemic. | 590 eligible participants (66 years old or above: 42 (7.1%)) | Younger people tended to have higher usage rates of electronic products than older people. 27 participants aged 66 or above indicated no increase of stress arising from staying at home. 30 participants aged 66 and above experienced minimal or none depressive symptoms (those aged 66 and above experienced much lower levels of depressive symptom). | 4 |
| Frey et al. (2020)^39^ | United States and 5 other countries | Impact of the COVID-19 Pandemic on the quality of life for women with ovarian cancer | Cross-sectional study | Evaluate the experience of women with ovarian cancer during the coronavirus disease 2019 pandemic. | 555 completed the survey. The median age was 58 years (range = 20–85; <65 = 74%; 65 = 26%). | Age less than 65 years and self-describing as immunocompromised were associated with higher levels of cancer worry, anxiety, and depression. | 4 |
| Frey et al. (2020)^40^ | United States | Gynecologic oncology care during the COVID-19 pandemic at three affiliated New York City hospitals | Prospective study | Investigate treatment patterns in gynecologic cancer care during the first two months of the COVID-19 pandemic at three affiliated New York City hospitals located in Brooklyn, Manhattan and Queens. | 302 patients with gynecologic cancer (65 years or more: 148 (49.0%)) | Patients 65 years and older were less likely to have treatment delays than younger patients. | 2b |
| García-Fernández et al. (2020)^97^ | Spain | Mental health in elderly Spanish people in times of COVID-19 outbreak | Cross-sectional study | Assess COVID-19 outbreak-related emotional symptoms, identify gender differences, and study the relationship between the emotional state and environmental features in the elderly. | One thousand six hundred thirty-nine (150 [9.2%] aged ≥60) participants | Older people have shown less emotional distress and they have not shown differences in anxiety levels during the peak of the pandemic when compared to the group under 60 years of age. The presence of economic losses as well as the increase in the use of anxiolytics was significantly associated with higher emotional distress in the elderly compared to the younger group. | 4 |
| García-Portilla et al. (2020)^98^ | Spain | Are older adults also at higher psychological risk from COVID-19? | Cross-sectional study | Describe the psychological responses of people aged ≥60 to this pandemic and lockdown situation and compare them with those under 60 years of age. | 1690 respondents aged ≥60 years, compared with 13,363 respondents under 60 years of age | Results on the DASS-21 scale (depressive, anxious, and stress response) demonstrated that females and males aged ≥60 years scored significantly lower on the three subscales, and they were less often considered probably a case than those aged <60 years. Concerning the IES (intrusive thoughts and avoidant behaviors), in both sexes, those ≥60 years scored significantly lower on the two subscales and fewer were classified as a probable case than respondents aged <60 years. Older women and men were considered a probable case of any emotional distress less frequently than younger women and men. | 4 |
| Giebel et al. (2020)^99^ | United Kingdom | A UK survey of COVID-19 related social support closures and their effects on older people, people with dementia, and carers | Cross-sectional study | Explore the impact of COVID‐19 public health measures on access to social support services and the effects of closures of services on the mental well‐being of older people and those affected by dementia. | 569 participants completed the survey (61 people with dementia, 219 current, 66 former carers, and 223 older adults). PLWD were on average 70±10 years of age (range 45-88), current carers: 61±13 (range 23-89), former carers: 64±14 (range 22-95), older adults who did not identify as living with dementia or being a carer for a person with dementia: 72±6 (range 65-90). | For those who had received social support services pre COVID, reductions in weekly social support service hours were significantly associated with reduced levels of well‐being in carers and older adults and anxiety in PLWD and older adults. Among older adults, being unable to access support services since COVID was significantly related to reductions in well‐being and increases in anxiety. COVID‐related closures were also significantly related to lower mental well‐being and higher levels of anxiety in older adults. The group with the highest proportion scoring above the cut off for both anxiety (33%) and depression (48%) were those living with dementia. In contrast, far fewer older adults achieved caseness (5% anxiety and 5% depression). | 4 |
| Giustino et al. (2020)^100^ | Italy | Physical Activity Levels and Related Energy Expenditure during COVID-19 Quarantine among the Sicilian Active Population: A Cross-Sectional Online Survey Study | Cross-sectional study | Measure the levels of PA expressed as energy expenditure (MET–minutes/week) among the physically active Sicilian population before and during the last seven days of COVID-19 quarantine. | 802 participants.  Elderly (>65 years) = 12 (1%) | Elderly (65 y and older) had the lowest physical activity levels among all age groups both before and during COVID-19 quarantine compared with younger participants (p<0.001 for differences among age groups). However, the elderly also had the smallest absolute decrease in physical activity level. | 4 |
| Goethals et al. (2020)^133^ | France | Impact of Home Quarantine on Physical Activity Among Older Adults Living at Home During the COVID-19 Pandemic: Qualitative Interview Study | Cross-sectional qualitative study | Evaluate the impact of this quarantine period on physical activity programs and on the physical and mental health of older adults. Discuss alternatives to physical activity programs that could be suggested for this population to avoid a sedentary lifestyle. | n=8 professionals (managers in charge of physical activity programs for older adults and sports trainers who run these physical activity programs) and n=6 older adults participating in a physical activity program | Because of the COVID-19 epidemic, attendance at physical activity workshops has declined. Despite the decline in their participation in group physical activities before the quarantine, older adults expressed the need to perform physical activity at home. | 4 |
| González-Sanguino et al. (2020)^101^ | Spain | Mental health consequences during the initial stage of the 2020 Coronavirus pandemic (COVID-19) in Spain | Cross-sectional study | Reporting the psychological impact of the Covid-19 outbreak in a sample of the Spanish population three weeks after the outbreak of the pandemic and declaration of the alarm state. | 3480 participants over 18 years of age. Aged 60-80 = 203 (5.8%) participants | Being older was significantly negatively related to depression (PHQ-2 difference = -0.902), anxiety (GAD-2 difference = -0.777) and PTSD (PCLC-2 difference = -0.291), compared to the 18-39 years old age group (p<0.001 for all). | 4 |
| Górnicka et al. (2020)^102^ | Poland | Dietary and lifestyle changes during covid-19 and the subsequent lockdowns among polish adults: A cross-sectional online survey PLifeCOVID-19 study | Cross-sectional study | Identify dietary changes patterns and their associations with socio-demographic factors, body mass index (BMI) before pandemic, and lifestyle behavior changes. We also examined the effect of lockdowns and restrictions linked to COVID-19 pandemic on dietary–lifestyle behaviors among adults in Poland. | n = 2381, ≥60 years = 6.1% (145) | 2.0% (13) increased consumption of healthy foods, 8.6% (108) were constant and 5.2 (24) increased the consumption of unhealthy foods. | 4 |
| Graham et al. (2020)^103^ | United Kingdom | Attitudes of ageing passengers to air travel since the coronavirus pandemic | Cross-sectional study | Analyse the attitudes of ageing passengers by assessing air travel plans in the next 12 months, examining the factors influencing future flying decisions, and investigating the impact of the coronavirus pandemic on perceived risks and experiences associated with flying. | 600 respondents aged ≥65 years old | Over 60% of ageing passengers are planning to travel by air in the next 12 months, although the nature of their trips may change. | 4 |
| Groarke et al. (2020)^104^ | United Kingdom | Loneliness in the UK during the COVID-19 pandemic: Cross-sectional results from the COVID-19 Psychological Wellbeing Study | Cross-sectional study | 1) Determine the rate of loneliness among adults in the UK during the early stages of the COVID-19 pandemic, and 2) Identify differences in sociodemographic, social, health, and COVID-19 specific factors between people with and without loneliness to determine the risk and protective factors for loneliness. | 1964 respondents aged 18 to 87 years; average age was 37.11 (≥65 years old: 3.1%) | The prevalence of loneliness decreased with age, with 18-24-year old’s having the highest frequency of loneliness (41%), whereas only 3% of people over 65 were classified as lonely. Relative to those over 65 years of age, younger adults were 4–5 times more likely to be lonely. | 4 |
| Gustavsson et al. (2020)^105^ | Sweden | Compliance to recommendations and mental health consequences among elderly in Sweden during the initial phase of the covid-19 pandemic—a cross-sectional online survey | Cross-sectional study | Explore the specific situation of people aged 70 and older in relation to COVID-19, more specifically, how people age 70 and older (a) perceive information and act on recommendations about the COVID-19 pandemic and (b) perceive how their mental health is affected by the current situation. | 1854 participants (mean age = 74.72) divided into age groups: 69-74 years old = 1058 (57.1%) and 75 years old and older= 796 (42.9%) | More than half of the respondents (60.8%) said they worry about their health during the COVID-19 pandemic. Respondents reported having trouble sleeping (23.5%), feeling depressed (38.8%), having difficulties concentrating (23.2%) and worrying about economic downturns (65.4%) and consequences to society (83.9%). 49.8% said the isolation makes them feel bad. | 4 |
| Haesebaert et al. (2020)^106^ | France | Who maintains good mental health in a locked-down country? A French nationwide online survey of 11,391 participants | Cross-sectional study | Investigate the sociodemographic and environmental determinants of wellbeing on the French population during lockdown due to the SARS-CoV-2 pandemic. | 11,391 participants, mean weighted age was 47.47 ±17.28 years (unweighted: 65-74=547 (4.80%), ≥75=81 (0.7%); weighted: 65-74=2469 (21.67%), ≥75=364 (3.20%)) | Age was positively correlated with wellbeing. Mean WEMWBS score (wellbeing) was 50.51±8.17, whereas for people aged 65-74 it was 52.61±16.15 and for people ≥75 it was 55.04±13.34. | 4 |
| Hamm et al. (2020)^42^ | United States | Experiences of American older adults with pre-existing depression during the beginnings of the covid-19 pandemic: A multicity, mixed-methods study | Longitudinal study | Determine the effect of the COVID-19 pandemic on the mental health of older adults with pre-existing major depressive disorder. | 73 community-living older adults (>60) with pre-existing major depressive disorder (MDD) (mean age 69.2 [SD 6]) | 53 participants described reduced quality of life from physical distancing, mostly due to decreased in-person social interaction and restrictions on leaving one’s home. However, mean (SD) change in the depression score on the PHQ-9 scale was –0.1 (4.9), p=0.80, which indicates compatibility with no observable change. | 2b |
| Hartley et al. (2020)^134^ | France | Les effets de confinement SARS-CoV-2 sur le sommeil : enquête en ligne au cours de la quatrième semaine de confinement. | Cross-sectional study | Explore the evolution of sleep during the SARS-CoV-2 quarantine period and define associated factors. | 1 777 participants, 4% were older than 70 years | Sleep deterioration was more pronounced in young people (54% of 25-34 years old vs. 22% of over 70 years old, p<0.0001). | 4 |
| Heid et al. (2020)^58^ | United States | Challenges Experienced by Older People During the Initial Months of the COVID-19 Pandemic | Cross-sectional study | Examine the extent to which older people are adhering to physical distancing mandates and the pandemic-related experiences that older people find most challenging. | 1,272 people (aged 64 and older), average age of 70.3 (SD = 6.65) | The majority of older people are staying away from public places, canceling physician’s appointments and medical treatments, spending less time with friends and relatives, canceling out-of-town trips, and changing plans to attend family gatherings. Most older adults (91%) reported that the COVID-19 pandemic has presented significant challenges. More specifically, they report difficulties across 8 domains: Social Relationships, Activity Restrictions, Psychological, Health, Financial, Global Environment, Death, and Home Care. The most frequently appraised challenges were constraints on social interactions (42.4%) and restrictions on activity (30.9%). | 4 |
| Heidinger et Richter (2020)^135^ | Austria | The Effect of COVID-19 on Loneliness in the Elderly. An Empirical Comparison of Pre-and Peri-Pandemic Loneliness in Community-Dwelling Elderly | Cross-sectional study at two different time points | Examines changes in loneliness in community-based elderly from Lower Austria, a county in Austria, comparing data from before and during the Covid-19 social distancing measures. | 888 individuals with a mean age of 73.0 (SD = 8.2) years (range 60-99 years) | Using the 6-Item De Jong Gierveld loneliness scale, participants reported mean scores of 1.61 (0.55) and 1.73 (0.60) before and during COVID-19, respectively, indicating an increase in loneliness (p=0.002). | 4 |
| Holingue et al. (2020)^59^ | United States | Mental distress during the COVID-19 pandemic among US adults without a pre-existing mental health condition: Findings from American trend panel survey | Cross-sectional study | Assess the frequency and risk and protective factors of psychological distress, during the beginning of the COVID-19 pandemic, within a nationally representative sample of adults in the US with no reported history of a pre-existing mental health condition. | 9687 individuals with no prior history of a mental health condition, divided into age categories | Old age (65+) was protective against levels of psychological distress. | 4 |
| Huang et al. (2020)^46^ | China | COVID-19 outbreak: The impact of stress on seizures in patients with epilepsy | Cross-sectional study | Evaluate the influence of the COVID-19 outbreak on seizures in patients with epilepsy (PWE) in the most severely affected area, Wuhan, and its surrounding cities. | 362 patients, n=6 (1.66%) patients were 60 years old or older | Among the 31 patients who had increase seizures during the outbreak, one patient was 60 years old or older. The 5 other participants of 60 years old or more had no seizure exacerbation. Age was significantly different between groups with and without seizure exacerbation. | 4 |
| Hyland et al. (2020)^107^ | Ireland | Anxiety and depression in the republic of Ireland during the covid‐19 pandemic | Cross-sectional study | Estimate the probable prevalence rates of generalized anxiety disorder (GAD) and depression and to identify sociodemographic risk factors associated with screening positive for GAD or depression. Our second aim was to determine if COVID‐19 related anxiety was highest amongst those in society at greatest risk of morality from COVID‐19. | (n = 1041, was 44.97 years (Mdn = 44.00, SD = 15.76, range 18–88), (65+ = 127) | Individuals aged 65 years old and older had lower odds of anxiety (Generalized Anxiety Disorder 7-item Scale score ≥10) or depression (Patient Health Questionnaire-9 score ≥10) than those aged 18-24 (p<0.001). COVID-19 related anxiety (“how anxious are you about the coronavirus COVID-19 pandemic?”; measured on a visual analogue scale) varied across age groups (p<0.001). Individuals aged 65 and older had higher scores than those aged 18-34 and 25-34 (mean [SD], 78 [22] vs. 61 [29] and 70 [26], respectively). | 4 |
| Islam et al. (2020)^60^ | United States | Examining COVID-19 Preventive Behaviors among Cancer Survivors in the United States: an analysis of the COVID-19 Impact Survey | Longitudinal study | Evaluate COVID-19–related preventive behaviors among cancer survivors using a nationally representative sample of U.S. adults, and further examine behaviors related to canceling or postponing activities, specifically doctor's appointments. | 10,760 respondents (60+: total=29.9%; cancer survivors=64.7%; never diagnosed with cancer=27.0%) | In age-adjusted analyses, we observed that cancer survivors aged 18 to 29 were more likely to cancel a doctor's appointment compared with those aged 60 years and above (PR: 1.83; 95% CI, 1.41–2.37). | 2b |
| Jacob et al. (2020)^108^ | United Kingdom | Challenges in the Practice of Sexual Medicine in the Time of COVID-19 in the United Kingdom | Cross-sectional epidemiological study | Investigate levels and correlates of sexual activity during COVID-19 self-isolation/social distancing in a sample of the UK public. | 868 individuals, 65-74 = 13.1% and 75 = 4.4% | Age was associated with fewer sexual activities per week, with means of 0.71 (1.44) for the group 65-74 and 0.26 (0.76) for the group ³75. | 4 |
| Jia et al. (2020)^109^ | United Kingdom | Mental health in the UK during the COVID-19 pandemic: cross-sectional analyses from a community cohort study | Cross-sectional study | Report the mental health sequelae of the COVID-19 pandemic in a UK cohort and examine modifiable and non- modifiable explanatory factors associated with mental health outcomes. | n=3097 adults aged ≥18 years; mean age 44±15 years; 65-74 years old: n=257 (8.3%), ≥75 years old: n=49 (1.6%) | Increased depression, anxiety and stress were associated with being younger. The mean values for all measures indicate levels that decrease with age. | 4 |
| Jiang et al. (2020)^75^ | China | Worries, Strategies and Confidence of Older Chinese Adults During the 2019 Novel Coronavirus Outbreak | Cross-sectional online surveys at two different time points | Investigate the mental state and attitudes of older Chinese adults during the COVID-19 epidemic. | First wave: 1148 respondents, the mean age was 39.8 (*SD =* 12.2) years, 72 (6.3%) were aged 60 years or above. Second wave: 470 respondents, the mean age was 42.8 (*SD =* 12.6) years, 42 (8.9%) were aged 60 years or above. | In the initial stage of the outbreak, results suggest a trend that older adults had fewer worries than young adults. Compared with old people, young people were more likely to see the importance of self‐prevention strategies. In the second wave data, the multiple group comparisons showed that older adults had higher self‐worries than the young group. Older adults in the second wave reported more worries than those in the first wave. | 4 |
| Karatzias et al. (2020)^136^ | Ireland | Posttraumatic Stress Symptoms and Associated Comorbidity During the COVID-19 Pandemic in Ireland: A Population-Based Study | Cross-sectional study | Determine rates of COVID-19–related posttraumatic stress disorder (PTSD) in the Irish general population, the level of  comorbidity with depression and anxiety, and the sociodemographic risk factors associated with COVID-19–related PTSD. | 1 041 participants, 12.2% were aged 65 years or above | Without controlling for anxiety and depression, participants in the three oldest age categories were less likely to screen positive for COVID-19–related PTSD compared to those in the youngest age category (i.e., 18–24 years). | 4 |
| Kivi et al. (2020)^110^ | Sweden | Up and about: Older adults' wellbeing during the COVID-19 pandemic in a Swedish longitudinal study | Longitudinal study | Investigate early effects of the COVID-19 pandemic related to (a) levels of worry, risk perception, and social distancing; (b) longitudinal effects on well-being; and (c) effects of worry, risk perception, and social distancing on well-being. | n = 1,071, aged 65–71 | 44.9% worried about health, 69.5% about societal consequences, 25.1% about financial consequences; 86.4% perceived a high societal risk, 42.3% a high risk of infection, and 71.2% reported high levels of social distancing. (b) Well-being remained stable (life satisfaction and loneliness) or even increased (self-rated health and financial satisfaction) in 2020 compared to previous years. (c) More worry about health and financial consequences was related to lower scores in all four well-being measures. Higher societal worry and more social distancing were related to higher well-being. | 2b |
| Klaiber et al. (2020)^65^ | Canada and United States | The ups and downs of daily life during COVID-19: Age differences in affect, stress, and positive events | Longitudinal study | Examine age differences in exposure and affective reactivity to daily stressors and positive events in the first several weeks of the COVID-19 outbreak. | 776 adults, aged 18-91 (mean age 45); ages 60-91 = 193 | Older adults were less concerned with harm to their emotional well-being, work goals, and finances. Middle-aged and older adults had higher positive affect and more daily positive events. Older adults had lower negative affect than both middle-aged and younger adults associated with fewer interpersonal conflicts, family, and work/school stressors but more “other” stressors. Older adults had fewer work/school/volunteer positive events but more remote positive social interactions, positive events in their social networks, nature events, and “other” types of positive events. | 2b |
| Knepple Carney et al. (2020)^61^ | United States | Age Moderates Perceived COVID-19 Disruption on Well-being | Cross-sectional study | Examine the Coronavirus Disease 2019 (COVID-19) disruption on well-being throughout adulthood. | 166 community-dwelling adults (mean age = 35.65; SD = 15.53; range = 18–79); 7.8% (n = 13) older adults (age range 60–79) | COVID-19 disruption appeared to have less of an effect on stress and negative affect with increased age. The effect of COVID-19 disruption on well-being does not vary between middle-aged (starting around the age of 50) and older adults. The current study found that when faced with a stressor, in this case a global pandemic, middle-aged and older adults may be better at regulating their emotions even when they perceive the stressor as disruptive. | 4 |
| Kotwal et al. (2020)^62^ | United States | Social Isolation and Loneliness Among San Francisco Bay Area Older Adults During the COVID-19 Shelter-in-Place Orders | Mixed-methods longitudinal phone-based survey  administered every 2 weeks | Investigate (1) experiences of  social isolation and loneliness during shelter-in-place orders,  and (2) unmet health needs related to changes in social  interactions. | 151 community-dwelling older adults aged 60 and older: mean age = 75.3±10.1 years | Participants reported social isolation in 40% of interviews. Socially isolated participants reported difficulty finding help with functional needs including bathing (20% vs 55%; P = .04). More than half (54%) of the participants reported worsened loneliness due to COVID-19 that was associated with worsened depression (62% vs 9%; P < .001) and anxiety (57% vs 9%; P < .001). Rates of loneliness improved on average by time since shelter-in-place orders (4–6 weeks: 46% vs 13–15 weeks: 27%; P = .009), however, loneliness persisted or worsened for a subgroup of participants. Open-ended responses revealed challenges faced by the subgroup experiencing persistent loneliness including poor emotional coping and discomfort with new technologies. | 2b |
| Krendl et al. (2020)^63^ | United States | The impact of sheltering-in-place during the COVID-19 pandemic on older adults' social and mental well-being | Longitudinal study | Examine whether social isolation due to the COVID-19 shelter-in-place orders was associated with greater loneliness and depression for older adults, and, if so, whether declines in social engagement or relationship strength moderated that relationship | 2019: 120 older adults (MAge = 74.68 years, SD = 7.13; 64 female)  ; Starting in mid-April 2020: 94 of the original participants (MAge = 75.20 years, SD = 6.86; 52 females; 1 withdrew and 2 interviews were lost) | Older adults reported higher depression and greater loneliness following the onset of the pandemic. Loneliness positively predicted depression. Perceived relationship strength, but not social engagement, moderated this relationship such that loneliness only predicted depression for individuals who became closer to their networks during the pandemic. The majority of older adults (79.3%; *N* = 69) said their social life had decreased/been negatively affected by COVID-19. Results revealed that older adults who felt less close to their social network during the pandemic (vs. prior to it) experienced increased depression irrespective of their loneliness. | 2b |
| Krok-Schoen et al. (2020)^166^ | United States and 13 other countries | Experiences of healthcare providers of older adults with cancer during the COVID-19 pandemic | Cross-sectional study | Examine clinical barriers to care, patient questions, and the overall experiences of oncology healthcare providers of older adults with cancer during the COVID-19 crisis. | 274 healthcare providers; the majority of respondents (68%) reported that older adults comprise over 50% of their patient volume | Barriers to care delivery included organizational challenges (e.g., delayed procedures/cancer care services), patients' access to resources and support (e.g., loss of income, limited home-based services), concerns for patients' mental and physical health (e.g., anxiety, depression, functional status, disease regression), and telehealth challenges (e.g., missed appointments, dissatisfaction). Respondents reported that older adults were asking about their health and cancer care (e.g., impact on prognosis and overall health, access to health care services) as well as access to basic needs and supports (e.g., transportation options, accesso to mental health services). | 4 |
| Kwegyir Tsiboe (2020)^47^ | Ghana | Describing the experiences of older persons with visual impairments during COVID-19 in rural Ghana. | Cross-sectional qualitative study | Describe the lived experiences among older persons with disability during the coronavirus pandemic in rural Ghana. | 20 participants, participants’ age ranged between 60 and 79 years | During the pandemic, care rendered to older persons with disabilities by their caregivers easily declined because of the lockdown measure. This made the participants suffer in profound loneliness and hunger and forced some to generate suicidal thoughts. On the other hand, the participants who lived with their family members were also kept indoors for several weeks to reduce their chances of contracting the virus. This was because participants’ family members lost confidence in the Ghanaian health-care system in protecting their older relatives. | 4 |
| Lara et al. (2020)^35^ | Spain | Neuropsychiatric symptoms and quality of life in Spanish Alzheimer’s disease patients during COVID-19 lockdown | Longitudinal study | Analyze the impact of the pandemic on these patients’ neuropsychiatric symptoms and their quality of life after five weeks of lockdown in Spain. | 20 with amnesic mild cognitive impairment (MCI) and 20 with mild Alzheimer’s disease (AD), with a mean age of 77.4 ± 5.25 | Significant changes were found regarding agitation, apathy and aberrant motor activity between T0 (4 weeks before; after 1 week of lockdown) and T1 (after 5 weeks of lockdown) and they were the most affected neuropsychiatric symptoms in patients. There were no differences in median quality‐of‐life scores across the 5 weeks of lockdown in patients or caregivers. 30% (12) of the patients and 40% (16) of the caregivers reported worse health condition scores. | 2b |
| Lee et al. (2020)^76^ | China | Higher psychogeriatric admissions in COVID-19 than in severe acute respiratory syndrome | Cross-sectional study | Examine whether psychogeriatric admissions increased after COVID-19, independent of seasonal variation; whether the increase was comparable with that seen in severe acute respiratory syndrome (SARS); and which factors were associated with such increase | All psychiatric admissions aged 65 or older in the 3 months before and  after COVID-19 (from November 2019 to April 2020) | Psychogeriatric admissions increased by 21.4% following the COVID-19 outbreak. This increase was not explained by seasonal variation and was greater and lasted longer than that in SARS. A rising trend in admissions for older adults living in residential care homes was observed. The increase in admissions was associated with fewer outpatient attendance, fewer home visits by nurses, and more older adults with dementia requiring inpatient care. | 4 |
| Li et al. (2020)^111^ | United Kingdom | Prevalence and predictors of general psychiatric disorders and loneliness during COVID-19 in the United Kingdom | Cross-sectional study | Explore the prevalence and predictors of general psychiatric disorders and loneliness in the United Kingdom with the first large-scale, nationally representative survey three months after the first diagnosis of COVID-19 in a developed country | 15,530 respondents. Age is divided into 5 categories: “18–30′′ (reference group), “31–40′′, “41–50′′, “51–65′′, and “over 65′′ | Prevalence of general psychiatric disorder: 65+ represents the lowest group, with 2.00 (2.69) (19.11 caseness ratio). Frequency of loneliness: 65+ represents the lowest group, with 4.11% who feel often lonely, 22.41% who feel sometimes lonely and 73.48 who feel never lonely. | 4 |
| Li (2020)^77^ | China | Psychosocial and coping responses towards 2019 coronavirus diseases (COVID-19): a cross-sectional study within the Chinese general population | Cross-sectional study | Determine the psychological status and post-traumatic stress symptoms (PTSD) among general population (except confirmed and suspected cases, and close contacts) and their association with the coping strategy types during the COVID-19 outbreak. | 1109 participants (≥60 years old: n=10 (0.90%)) | Three participants aged ≥60 years reported psychiatric disorders, with GHQ-28 scale scored 5 or more (subscales: somatic symptoms, anxiety and insomnia, social dysfunction and severe depression). Seven participants aged ≥60 years were in high post-traumatic stress symptoms level, with IES-R scale scored 20 or more (subscales: intrusion, avoidance and hyperarousal). Younger groups (30-50) were at higher risk psychiatric disorders toward this outbreak. | 4 |
| Li et al. (2020)^78^ | China | Prevalence and factors for anxiety during the coronavirus disease 2019 (COVID-19) epidemic among the teachers in China | Cross-sectional study | Assess the prevalence of anxiety and explore its factors during the Coronavirus Disease 2019(COVID-19) epidemic among the teachers in China. | 88,611 teachers aged 18 to 100 years, mean age was 36.22±9.02 years | The highest prevalence of anxiety was 14.06% (SE 2.51%) with age between 60 and 100 years in men, and14.70% (SE 0.56%) with age between 50 and 60 years in women. The lowest prevalence of anxiety was 12.36% (SE 2.89%) found in participants with the age between 40 and 50 years in men, and 11.76% (SE4.30%) with age of between 60 and 100 years in women. For minimal anxiety, the proportion of participants with age of 60 to 100 years was the highest (52.67%) and 30 to 40 years was the lowest (47.27%); for mild anxiety, age of 30 to 40 years was the highest (38.73%) and 60 to 100 years was the lowest (33.74%); for moderate anxiety, age of 30 to 40 years was the highest (9.50%) and 60 to 100 years was the lowest (7.41%); for severe anxiety, age of 60 to 100 years was the highest (6.17%) and 18 to 30 years was the lowest (4.07%). There was a significant proportion of participants aged between 60 and 100 years who presented severe anxiety level. | 4 |
| Liu et al. (2020)^79^ | China | Psychological status and behavior changes of the public during the COVID-19 epidemic in China | Cross-sectional study | Investigate the psychological status and behavior changes among ordinary Chinese people during the COVID-19 epidemic and evaluated whether these factors were related to the spread of the disease. | 608 valid questionnaires included in our study, 153 respondents did not complete the SCL-90, and only 455 respondents completed all survey scales. 70 = 5 (0.8%) | No participant of 70 had depression or psychology abnormality. | 4 |
| López et al. (2020)^112^ | Spain | Psychological well-being among older adults during the covid-19 outbreak: A comparative study of the young–old and the old–old adults | Cross-sectional study | Investigated the psychological well-being experienced by young–old and old–old adults during the COVID-19 crisis and the variables associated. | 878 community-dwelling older adults from Spain (626 from 60 to 70 and 252 from 71 to 80 years old) | Young–old experienced more personal growth, but they didn’t experience more purpose in life than old–old. Old–old experienced more gratitude, and more resilience. All personal resources, including more resilience and gratitude and less experiential avoidance, were significantly associated with more purpose in life. | 4 |
| Malta et al. (2020)^149^ | Brazil | The COVID-19 Pandemic and changes in adult Brazilian lifestyles: a cross-sectional study, 2020 | Cross-sectional study | Describe lifestyle changes with regard to consumption of tobacco and alcohol, food intake and physical activity, in the period of social restriction resulting from the COVID-19 pandemic. | 45,161 individuals aged 18 years or more (≥60 years old: 20.3%) | An increase of 5 cigarettes per day was more prevalent among young adults aged 18-29, compared to individuals aged 50 and over while an increase of 10 cigarettes and more than 20 cigarettes a day was similar in all age groups. Lowest prevalence of alcohol consumption was found among the elderly. No differences between age groups were found in the frequency of healthy food consumption, although their consumption remained higher among the elderly (60 years old or over). No increase in the frequency of consumption of unhealthy food was found among the elderly. Among the elderly (60 years old or over), sufficient physical activity fell from 30.4% to 14.2%. Despite the elderly being the group with the highest average time spent watching TV, they accounted for the lowest average increase, just one hour more, during the period of social restriction. | 4 |
| Mann et al. (2020)^66^ | United States | Personal economic anxiety in response to COVID-19 | Cross-sectional study | Examine demographic and individual correlates of anxiety about financial hardship on March 17th, 2020, the day after historic stock market drops in response to the emerging COVID-19crisis. | 513 adults; the age of participants spanned 20 to 79 years (mean= 38.55, SD= 11.91) | Age was negatively correlated with economic anxiety. Younger adults tended to report greater anxiety than older adults. | 4 |
| McAndrew et al. (2020)^113^ | Ireland | Impact of initial COVID-19 restrictions on psychiatry presentations to the Emergency Department of a large academic teaching hospital | Cross-sectional study | Determine if the initial COVID-19 societal restrictions, introduced in Ireland in March 2020, impacted on the number and nature of psychiatry presentations to the emergency department (ED) of a large academic teaching hospital. | Number of psychiatry presentations to the emergency department | Compared with the reference period from 2018 and 2019, the age distribution of psychiatry presentations to ED during COVID-19 was different (p=0.002). The total number of cases for individuals aged 60 years or older was 23 in 2018/2019 (mean) and 14 during COVID-19. | 4 |
| Moen et al. (2020)^72^ | United States | Disparate Disruptions: Intersectional COVID-19 Employment Effects by Age, Gender, Education, and Race/Ethnicity | Cross-sectional survey | Examine disparities in the employment effects of COVID-19 across intersecting subgroups in the United States. | 116,649 men and women aged 60 years and older (60-69: 58,984 / 70+: 57,665) | From January to April 2020: in women, unemployment increased by 4.6% and by 1.5% for 60-69 y and 70 years and older, respectively; in men, unemployment increased by 4.1% and by 1.7% for 60-69 years and 70 years and older, respectively. Of note, this increase was the lowest compared with other age groups (individuals aged 20-59 y). Retired women decreased by 0.4% and increased by 0.8% for 60-69 y and 70 years and older, respectively; Retired men increased by 0.5% and by 2.7% for 60-69 y and 70 years and older, respectively | 4 |
| Neill et al. (2020)^154^ | Australia | Alcohol use in Australia during the early days of the COVID-19 pandemic: Initial results from the COLLATE project | Cross-sectional study | Examine what predisposing (distal) and pandemic-related (proximal) factors were associated with increased drinking in the wake of the COVID-19 pandemic. | 4462 individuals, n=317 (6.1%) were aged 65 years or older | Those aged 65 years or older were less likely to report an increase in drinking (P< 0.001). | 4 |
| Newby et al. (2020)^151^ | Australia | Acute mental health responses during the COVID-19 pandemic in Australia | Cross-sectional study | 1) Provide the first snapshot of the mental health of the general community during the initial COVID-19 outbreak (and enforcement of social distancing laws) in Australia. 2) Explore the relationship between specific demographic and sample characteristics with depression, anxiety and stress, to identify factors that are associated with increased vulnerability for poorer mental health during the COVID-19 pandemic. | 5071 participants ranged in age from 18 to over 75 (65-74 years old: n=497 (9.8%); 75+ years old: n=51 (1.0%)) | Older age was a predictor of lower depression, lower anxiety and lower stress. | 4 |
| Niedzwiedz et al. (2020)^114^ | United Kingdom | Mental health and health behaviours before and during the initial phase of the COVID-19 lockdown: longitudinal analyses of the UK Household Longitudinal Study | Longitudinal study | Examine trends in mental health and health behaviours in the UK before and during the initial phase of the COVID-19 lockdown and differences across population subgroups. | Wave 7 (2015–2017): 65+= 5862 (23.2%); Wave 8 (2016–2018): 65+= 5826 (23.4%); Wave 9 (2017–2019): 65+= 5524 (23.6%); COVID-19 Wave (April 2020): 65+= 2934 (22.7%) | Increases were noted in psychological distress, binge drinking and alcohol frequency in 65+ people. However, younger people experienced the greatest relative increase in poor mental health. | 2b |
| Nimrod (2020)^144^ | Israel | Changes in Internet Use When Coping With Stress: Older Adults During the COVID-19 Pandemic | Cross-sectional study | Explore older people’s use of the Internet for coping with stress posed by the COVID-19 pandemic. | 407 Internet users from 60 to 84y old (mean = 69.14±5.14) | Participants’ reports indicated a significant increase in Internet use following the onset of the pandemic. Stress levels were moderate-to-high and participants appeared more worried about others than about themselves. Moderate levels of subjective well-being were founded. Significant positive associations were found between stress and increase in Internet use for interpersonal communication and online errands. Linear regression analysis revealed a significant negative association between stress and subjective wellbeing, but it was only increased Internet use for leisure that associated significantly with enhanced wellbeing. | 4 |
| Ntsama Essomba et al. (2020)^163^ | Cameroon | The Follow-Up and Well-Being of Geriatric Outpatients During COVID-19 Pandemic in Cameroon: Insights From the Yaounde Central Hospital | Cross-sectional study (retrospective) | Examine the effects of the COVID-19 pandemic on the follow-up and well-being of older outpatients seen at geriatric consultation in Cameroon. | 30 participants aged 65 and over. The median age of participants was 74, with 40% (n = 12) aged ≥80 years. | Most patients (73%, n= 22) did not attend their appointment because of fear of being infected by SARS-CoV-2 at hospital. Approximately 23% (n= 7) of participants reported a decline of their functional status since the last geriatric visit. Loss of appetite and weight loss were both reported in 30% (n= 9) of patients. Half of participants (n= 15) self-rated their health status as bad. | 4 |
| Nwachukwu et al. (2020)^67^ | Canada | Covid-19 pandemic: Age-related differences in measures of stress, anxiety and depression in Canada | Cross-sectional study | Examine the evidence for the impact of age on stress, anxiety, and depression levels in the COVID-19 pandemic from the perspective of a Canadian cohort with the goal of informing policy planning in relation to age-appropriate mental health supports and resource allocations during thisCOVID-19 pandemic period. | 8267 individuals, 762 (9.2%) identified as over 60 years | The prevalence rates for moderate/high stress, likely generalized anxiety disorder, and likely major depressive disorder as well as the mean scores on the Perceived Stress Scale (PSS), the Generalized Anxiety Disorder 7-item (GAD-7) scale, and the Patient Health Questionnaire-9 (PHQ-9) were highest amongst those aged under 25 years, and lowest amongst those over 60 years. | 4 |
| Ogden (2020)^115^ | United Kingdom | The passage of time during the UK Covid-19 lockdown | Cross-sectional study | Establish how the passage of time was experienced during the Covid-19 lockdown in the UK. | 604 participants, mean age of 34.88 (14.24), >60 (7.90%) | For both POTJ-day and POTJ-week, being over the age of 60 was associated with a significant slowing of the passage of time, people under the age of 60 appear to experience the passage of time comparably. | 4 |
| Owen et al. (2020)^152^ | Australia | Poor appetite and overeating reported by adults in Australia during the COVID-19 pandemic: A population-based study | Cross-sectional study | Identify self-reports of being bothered by poor appetite or overeating in adults in Australia during the COVID-19 lockdown and learn whether this differs by age, gender or personal and living circumstances. | 13 829 Australian residents aged 18 years or over; 60-69: 2833 (20.5%), 70+: 1447 (10.5%) | Increasing age was incrementally associated with decreasing odds of being bothered by poor appetite or overeating in the previous fortnight. | 4 |
| Ozamiz-Etxebarria et al. (2020)^116^ | Spain | Stress, anxiety, and depression levels in the initial stage of the COVID-19 outbreak in a population sample in the northern Spain | Cross-sectional exploratory-descriptive study | Understand the levels of psychological impact, anxiety, depression, and stress, among other variables, in the initial stage of the COVID-19 outbreak. | 976 individuals from the Basque Autonomous Community. 78 had >61 years of age. | The mean levels of symptoms (depression, anxiety and stress levels) in the three dimensions were lowest in individuals 61 years and older. | 4 |
| Pahayahay et al. (2020)^167^ | Canada and many others | What Media Helps, What Media Hurts: A Mixed Methods Survey Study of Coping with COVID-19 Using the Media Repertoire Framework and the Appraisal Theory of Stress | Cross-sectional study | Investigate the relation between subjective stress and changes in the pattern of media use in the immediate aftermath of the COVID-19 lockdowns. | 685 participants (older than 65 years: n=89 (12%)) | Majority of participants older than 65 years are slightly worried by COVID-19 and some were very stressed. A higher proportion of young respondents considered their mental health as poor or could be better. Interestingly, the differences in game use of those older than 65years were not significantly different from those who were younger than 25 years or those aged 25-34years. Even the mean ranks of game use in the older than 65 years category were higher than those aged 55-65 years. | 4 |
| Parlapani et al. (2020)^117^ | Greece | Intolerance of Uncertainty and Loneliness in Older Adults During the COVID-19 Pandemic | Cross-sectional study | Investigate the psychological response of older adults during the acute phase of the pandemic in Greece. | 103 participants over 60y old (mean age=69.85±5.26) | 81.6% of the participants reported moderate to severe depressive symptoms, 84.5% reported moderate to severe anxiety symptoms and 37.9% reported disrupted sleep. Moreover, a total of 35 participants reported suicidal ideation. Participants living alone showed higher levels of loneliness (p = .004) compared with participants living together with their family or a caregiver. | 4 |
| Parlapani et al. (2020)^118^ | Greece | Psychological and Behavioral Responses to the COVID-19 Pandemic in Greece | Cross-sectional study | Explore COVID-19-related fear, depressive and anxiety symptoms, social responsibility, and behavioral responses during the COVID-19 pandemic in Greece. | 3 029 participants | With regard to age, younger participants, under the age of 30, displayed less fear of COVID-19 and reported less severe depressive symptoms compared with older participants (≥61). Contrary to fear and depressive symptoms, anxiety symptoms’ severity in younger participants did not differ significantly compared with older ones. | 4 |
| Philip et al. (2020)^44^ | United Kingdom | Respiratory patient experience of measures to reduce risk of COVID-19: Findings from a descriptive cross-sectional UK wide survey | Cross-sectional study | Assess the experience of people with long-term respiratory conditions regarding the impact of measures to reduce risk of COVID-19. | 9515 people with self-reported long-term respiratory conditions, age ranges from ≤17 years to 80 years and above; 60-69 years old: n=1072; 70-79 years old: n=538; 80 and above: n=72 | Levels of anxiety about COVID-19 decreased slightly with age. Those aged 80 and above had the lowest level of anxiety. Older age groups were more likely to report coping well with 47% of 70–79 years old, steadily decreasing to 17% in the 18–29 years old group (Kruskal-Wallis p<0.001). | 4 |
| Picaza Gorrochategi et al. (2020)^119^ | Spain | Stress, anxiety, and depression in people aged over 60 in the covid-19 outbreak in a sample collected in northern Spain | Exploratory-descriptive cross-sectional methodology | Measure the levels of stress, anxiety, and depression in older people and to also explore the relationships between these variables and chronic diseases | 290 people from the Basque Autonomous Community (BAC), of whom 62.1% (n = 180) were women. 36.6% (n = 106) had between 60 and 65 years of age and 63.4% (n = 184) were over 66 years of age (M= 66.36; DT= 5.03). 32.8% (n = 95) indicated having a chronic disease. | The majority of participants did not report stress (60-65: mild (9.5%), moderate (2.9%), severe (1.0%) and extremely severe (0.0%); 66: mild (6.9%), moderate (3.8%), severe (0.7%) and extremely severe (0.0%)), anxiety (60-65: mild (4.7%), moderate (11.3%), severe (0.9%) and extremely severe (0.9%); 66: mild (4.3%), moderate (6.0%), severe (0.0%) and extremely severe (0.5%)), or depression (60-65: mild (2.4%), moderate (3.4%), severe (1%) and extremely severe (0.3%); 66: mild (5.9%), moderate (4.8%), severe (0.3%) and extremely severe (0.3%)). | 4 |
| Pieh et al. (2020)^120^ | Austria | The effect of age, gender, income, work, and physical activity on mental health during coronavirus disease (COVID-19) lockdown in Austria | Cross-sectional study | Evaluate mental health in a representative adult sample in Austria after 4 weeks of lookdown considering relevant influencing factors such as age, gender, income, and job situation. | 1005 participants (65+ = 133 (13.2%)) | Individuals being 65+ years were less burdened in all scales (quality of life, well-being, perceived stress, depressive symptoms, anxiety, sleep quality) considering mean scores. | 4 |
| Pierce et al. (2020)^121^ | United Kingdom | Mental health before and during the COVID-19 pandemic: a longitudinal probability sample survey of the UK population | Longitudinal cohort study | Examine changes in adult mental health in the UK population before and during the lockdown. | 17 452 participants (≥70 = 2633) | Mental health changes (assessed using the 12-item General Health Questionnaire [GHQ-12]) varied across age groups during the COVID-19 pandemic compared with preceding trends (p<0.0001). However, within-group change of individuals aged ≥70 years old was consistent with little to no change; the adjusted mean change in GHQ-12 score was 0.17 (95%CI, -0.33 to 0.68). Mean scores were higher in younger age groups than in older ones. | 2b |
| Ping et al. (2020)^80^ | China | Evaluation of health-related quality of life using EQ-5D in China during the COVID-19 pandemic | Cross-sectional study | Know about the impact of the COVID-19 epidemic on the health-related quality of life (HRQOL) of living using EQ-5D in general population in China. | 1139 (60+ = 76 (6.7%)) | Older age was associated with lower EQ-5D index score (mobility, self-care, usual activities, pain/discomfort, and anxiety/depression) and lower VAS score (overall health) (lower scores = worst health status). Problems were reported in the following dimensions: anxiety/depression (23.7%), pain/discomfort (52.6%), usual activities (7.9%), self-care (0%) and mobility (13.2%). | 4 |
| Pinto et al. (2020)^122^ | Portugal | Sleep quality in times of Covid-19 pandemic | Cross-sectional study | Evaluate the sleep quality of respiratory patients during the COVID-19 pandemic lockdown. | 365 participants (mean age: 63.9 ± 13.1) | Older age (specifically over 65 years old) was a protective factor for reporting difficulties falling asleep, waking up too early and non-restorative sleep: compared with participants 65 years or younger, participants over 65 years old were less likely to reported difficulties falling asleep (OR= 0.54; 96%CI, 0.33 to 0.88), less likely to report non-restorative sleep (OR= 0.57; 95%CI, 0.35 to 0.95) and less likely to wake up too early in the morning (OR= 0.41; 95%C1, 0.28 to 0.67). | 4 |
| Qin et al. (2020)^81^ | China | Physical activity, screen time, and emotional well-being during the 2019 novel coronavirus outbreak in China | Cross-sectional study | Evaluate the effects of the COVID-19 lock down on lifestyle in China during the initial stage of the pandemic. | 12,107 participants aged 18–80 years were included (60 =184 (1.5%)) | Levels of physical activity during home quarantine: 60 = vigorous (30.4%), moderate (28.3%) and light (41.3%).  Lower prevalence of insufficient physical activity and less screen time were observed in those aged 55–59 years and over 60 years old during home quarantine induced by COVID-19. | 4 |
| Ring et al. (2020)^145^ | Israel | The moderating role of subjective nearness-to-death in the association between health worries and death anxieties from COVID-19 | Cross-sectional study | Examine whether subjective nearness-to-death moderated the association between health worries and death anxiety due to the COVID-19 outbreak among older adults in Israel. | 277 older adults; the age range of the participants was 60 to 92 years (M=69.59, SD=6.72). | Subjective nearness-to-death moderated the association between health worries and death anxiety among older adults during the pandemic period. Under the condition of close nearness to death and a high level of COVID-19 health worries, death anxiety is high. Additionally, those who felt that death was far away, reported a lower level of anxiety about their death. | 4 |
| Rogers et al. (2020)^137^ | United Kingdom | Behavioral Change Towards Reduced Intensity Physical Activity Is Disproportionately Prevalent Among Adults with Serious Health Issues or Self-Perception of High Risk During the UK COVID-19 Lockdown | Cross-sectional study | Assess whether lockdown had a disproportionate impact on physical activity behavior in groups who were, or who perceived themselves to be, at heightened risk from COVID-19. | 9 190 participants, n=1 062 (11.56%) were aged 70+ | Compared to the oldest age group (70+), younger age groups were significantly more likely to have changed and to be doing either more, or indeed less intense PA since the lockdown began. | 4 |
| Rolland et al. (2020)^123^ | France | Global Changes and Factors of Increase in Caloric/Salty Food Intake, Screen Use, and Substance Use During the Early COVID-19 Containment Phase in the General Population in France: Survey Study | Cross-sectional study | Assess the global changes and factors of increase in addiction-related habits during the early COVID-19containment phase in France. | 11,391 participants with a weighted mean age of 47.47 years (SD 17.28); 65-74: n=2469 (21.67%) [weighted], ≥75: n=364 (3.20%) [weighted] | Older ages (≥65) were not a factor of increased in caloric/salty food intake, screen use, tobacco use, alcohol use and cannabis use. Age less than 29 years was a factor of increase. | 4 |
| Romero et al. (2020)^124^ | Spain | COVID-19 psychological impact in 3109 healthcare workers in Spain: The PSIMCOV group | Cross-sectional study | Assess the psychological impact of the COVID-19 pandemic in Spanish healthcare workers. | 3109 healthcare workers, mean age=45.14±6.48; 60-69 years: n=334 (10.7%), ≥70 years: n=8 (0.3%) | The least stressed respondents were asymptomatic workers, Psychological Stress and Adaptation at work Score (PSAS), 41.3 (15.4); p< 0.001, as well as those above 60 years old, PSAS, 37.6 (16); p< 0.001. | 4 |
| Romito et al. (2020)^125^ | Italy | Psychological Distress in Outpatients with Lymphoma During the COVID-19 Pandemic | Cross-sectional study | Assess the psychological status of outpatients receiving anti-neoplastic treatment for lymphoproliferative diseases during lockdown in a non-COVID Cancer Center Institute in southern Italy, pursuing the following aims: (i) to measure the levels of post-traumatic symptoms, depression, and anxiety during the pandemic; and (ii) to investigate the perception of the risk of potential nosocomial infection. | 77 outpatients, mean age was 56.6 (range 22–85) | When the age groups (18–50; 50–70 and >70) were correlated with the dependent variables, higher levels of post-traumatic stress disorder (IES-R) were found in the younger age group (namely 18–50) (r=0.43, p=0.03). Women and younger patients were found to be more vulnerable to anxiety and post-traumatic stress disorder. | 4 |
| Shrira et al. (2020)^146^ | Israel | Covid-19-related loneliness and psychiatric symptoms among older adults: The buffering role of subjective age | Cross-sectional study | Examine whether subjective age moderated the relationship between loneliness due to the COVID-19 pandemic and psychiatric symptoms. | 277 older adults (mean age = 69.58 ± 6.72), range 60–92 | Respondents felt on average younger than their age. Although the mean loneliness and psychiatric symptom scores were generally low, there was marked variability among respondents. All respondents reported changing at least one behavior due to the pandemic (ranging from buying more food and water than usual: n = 91, 32.9%, to going out less frequently: n = 246, 88.8%). | 4 |
| Sigorski et al. (2020)^41^ | Poland | Impact of COVID-19 on anxiety levels among patients with cancer actively treated with systemic therapy | Prospective study | Assess the relationship between the level of anxiety caused by a neoplasm and the threat of coronavirus infection among patients with cancer actively treated with systemic therapy during the COVID-19 pandemic. Additionally, we searched for clinical factors associated with a higher level of anxiety. | 306 actively treated patients with cancer, n=112 (41.50%) patients were over 65 years of age | There were no statistically significant differences in fear/anxiety of COVID-19 between younger and older patients. Fear and anxiety associated with cancer was higher for those aged 65 years and under than for those older than 65 years (6.73±2.96 vs 5.66±3.24; p=0.007). | 2b |
| Smith et al. (2020)^126^ | United Kingdom | Correlates of symptoms of anxiety and depression and mental wellbeing associated with COVID-19: a cross-sectional study of UK-based respondents | Cross-sectional study | Assess the impact of COVID-19 self-isolation/social distancing on mental health, and potential correlates, among a sample of the UK population. | 932 adults (65-74 years old (13.9%) and 75 years old and more (4.4%)) | Younger age was correlated with poor mental health. | 4 |
| Sorokin et al. (2020)^164^ | Russia | Structure of anxiety associated with Сovid-19 pandemic: The online survey results | Cross-sectional study | Reveal the structure of anxiety in the population during the epidemic period and to identify the most vulnerable social groups which were most in need of psychological and/or psychiatric help. | 1957 participants including 58 individuals aged 60-78 years | 40% of participants over 60 years of age were concerned about financial difficulties caused by the pandemic, which was the highest among age groups. Also, 12.1% of the individuals aged 60 or over were concerned about risk associated with social isolation, but this prevalence was lower than individuals aged 18-20 (29%) and 21-30 (20%). | 4 |
| Stanton et al. (2020)^153^ | Australia | Depression, anxiety and stress during COVID-19: Associations with changes in physical activity, sleep, tobacco and alcohol use in Australian adults | Cross-sectional study | Examine associations between depression, anxiety and stress and changes in health behaviors, including physical activity, sleep, smoking and alcohol use subsequent to the onset of COVID-19 and the implementation of social isolation rules in Australia. | 1491 people (mean age 50.5 ± 14.9 years, 999 female) | People aged more than 65 years old had lower depression, anxiety and stress scores than other age groups. | 4 |
| Stickley et al. (2020)^140^ | Japan | Loneliness and COVID-19 preventive behaviours among Japanese adults | Cross-sectional study | Examine the association between loneliness and COVID-19 preventive behaviours among adults in Japan. | 2000 individuals (674 had more than 60 years old) | 29.8% of individuals aged more than 60 years old were feeling lonely. Younger individuals were feeling lonelier. | 4 |
| Stylianou et al. (2020)^165^ | Cyprus | Mental Health Disorders During the COVID-19 Outbreak in Cyprus | Cross-sectional study | Assess the mental health burden of the Cypriot population during the outbreak and to explore the potential influence factors. | 216 volunteers (n=9 (4.17%) were 65 years old or more) | No participants aged above 65 years of age have reported major generalized anxiety disorder. The youngest age group (≤21) had the highest prevalence (21.74%) of major depressive symptoms, and the participants >65 did not have any. | 4 |
| Sun et al. (2020)^82^ | China | Influencing factors of understanding covid-19 risks and coping behaviors among the elderly population | Cross-sectional study | Understand the influencing factors of COVID-19-related risks and coping behaviors of elderly individuals with respect to COVID-19 and to provide a basis for taking corresponding protective measures. | 508 elderly individuals over 60 years old; 60-70 years old: n=239 (47.0%), 71-80 years old: n=185 (36.4%), over 80 years old: n=84 (16.5%) | 76.77% of participants frequently or always eat a balanced diet, quit drinking alcohol, and maintain adequate sleep and rest times. | 4 |
| Suzuki et al. (2020)^141^ | Japan | Physical Activity Changes and Its Risk Factors among Community-Dwelling Japanese Older Adults during the COVID-19 Epidemic: Associations with Subjective Well-Being and Health-Related Quality of Life | Longitudinal study | Assess how public health restrictions impact PA, subjective well-being (SWB), and health-related quality of life (HRQoL) of community-dwelling elderly. Also, investigate risk factors that lead to a decline in PA. | 165 Community-Dwelling Japanese Older Adults aged over 65 years old (mean age: 78.5 ± 8.0 years). | During COVID-19 restrictions, 78 individuals (47%) were less active, 38 (23%) were more active and 49 (30%) maintained their physical activity level compared with before. Subjective well-being decreased in the less active group only during COVID-19 compared with before (-2.5 points; p<0.01). SWB scores significantly decreased in the less active group but this was not seen in the more or equally active group. HRQoL scores were reduced by COVID-19 restrictions regardless of changes in PA. | 2b |
| Szabo et al. (2020)^138^ | Hungary | Attitudes Toward COVID-19 and Stress Levels in Hungary: Effects of Age, Perceived Health Status, and Gender | Cross-sectional study | Understand how Hungarians cope with the COVID-19 situation. | 1,552 volunteers (n=235 were 60 years old or more) | Older adults perceived the threat higher than middle-aged adults but did not differ from young adults. Older adults reported less stress than the younger groups, and middle-aged adults reported less stress than the young adults. | 4 |
| Tian et al. (2020)^83^ | China | Psychological symptoms of ordinary Chinese citizens based on SCL-90 during the level I emergency response to COVID-19 | Cross-sectional observational study | Investigate the severity of psychological symptoms of ordinary Chinese citizens during the Level I Emergency Response period through the SCL-90. | 1060 individuals (36 had more than 60 years old (3.4%)) | The overall index of psychological symptoms (90 items assessing somatization, obsessive-compulsive, interpersonal sensitivity, depression, anxiety, hostility, phobic anxiety, paranoid ideation, psychoticism) varied across age groups (p<0.001) and people aged more than 60 years old had the highest score on this scale (older ordinary people have more severe psychological symptoms). | 4 |
| Van der Roest et al. (2020)^38^ | Netherlands | The Impact of COVID-19 Measures on Well-Being of Older Long-Term Care Facility Residents in the Netherlands | Cross-sectional study | Gain insight into the consequences of COVID-19 measures on loneliness, mood, and behavioral problems in residents in Dutch long-term care facilities. | 193 residents without severe cognitive impairment (CI), 1609 family members of residents with and without CI, and 811 care professionals | Loneliness was reported by 149 (77%) residents: 50% perceived themselves as moderately,16% as strongly, and 11% as very strongly lonely. Relatives and staff classified respondents as not lonely (14%;19%, respectively), moderately (50%; 34%), strongly (25%; 31%), and very lonely (11%; 16%). Staff classified residents without CI more lonely than residents with CI. 51% of residents had poor mental health. Only 27% of relatives reported no change in residents’ mood status. Happiness was less often, and sadness was more often reported by family of residents without CI than with CI. More than half of the staff reported an increase in severity of agitation, depression, anxiety, and irritability. | 4 |
| van der Velden et al. (2020)^127^ | Netherlands | Anxiety and depression symptoms, and lack of emotional support among the general population before and during the COVID-19 pandemic. A prospective national study on prevalence and risk factors | Prospective study | Compare the prevalence of high Anxiety and Depression Symptom (ADS) levels and lack of Emotional Support (ES) during the in the Netherlands during March 2020, with high ADS levels and lack of ES before the COVID-19. Also, examine to what extent specific subgroups are at risk for high ADS levels and lack of ES compared to “normal” circumstances. | 3983 individuals | With respect to pre-out-break high ADS levels, older respondents had less often high ADS levels than the youngest group of respondents. 65 y and older individuals compared with 18-34 were less likely to experience high ADS, both before and during COVID-19. | 2b |
| vanTilburg et al. (2020)^128^ | Netherlands | Loneliness and mental health during the COVID-19 pandemic: A study among Dutch older adults | Longitudinal study | Evaluate the impact of reduction in the frequency of social contacts, personal losses, and the experience of general threats on society well-being. | 1,679 Dutch community-dwelling participants aged 65–102 years (M age=73) | Compared with 7 months before, the respondents were more socially and, especially, emotionally lonely during the pandemic. Mental health remained roughly stable. The item “I miss having people around me” increased by 34 percentage points. Many reported being personally affected by the loss of social contact, and fewer by loss of their own or someone else’s work or activities, or by being less frequently outdoors. Some respondents indicated that they had a personal, domestic, or social need that was not met. | 2b |
| Venugopal et al. (2020)^156^ | India | Status of mental health and its associated factors among the general populace of India during COVID-19 pandemic | Cross-sectional survey | Examine the mental health status of the general  population to understand the psychological impact of COVID-19  lockdown on individuals. | 453 participants including 32 (7.06%) participants older than 60 years. | About 40.63% of the elderly population (above 60 years old) was under severe physiological distress. The average general health score of different agegroups was found in the order of older people (above 60) > mid-age (31-60) > Adults (18-30) (the lower score, better the mental health status, and vice versa). | 4 |
| Wammes et al. (2020)^139^ | Netherlands | Evaluating Perspectives of Relatives of Nursing Home Residents on the Nursing Home Visiting Restrictions During the COVID-19 Crisis: A Dutch Cross-Sectional Survey Study | Cross-sectional study | Capture perspectives from the relatives of nursing home residents on nursing home visiting restrictions. | 1997 respondents, mean age 60.1 years (range 20-97) | Most relatives selected loneliness (76%), sadness (66%), and loss of quality of life (62%) as potential adverse effects on nursing home residents. Respondents specified in the “other” option that adverse effects also included increase of cognitive impairment, depressive symptoms, insufficient personal care (e.g., care of hair and nails), and especially for persons with dementia, alienation from their social network. | 4 |
| Wang et al. (2020)^129^ | Germany | The impact and consequences of sars-cov-2 pandemic on a single university dermatology outpatient clinic in Germany | Retrospective study | Investigate the impact of COVID-19 pandemic on the university dermatology outpatient clinic (UDOC) of the Technical University of Munich, Germany. | All consultations at the clinic in 2019 (2043 consultations) and 2020 (2979 consultations) | Patients aged 85 years or older were more likely to miss their consultation in 2020 compared with other age groups (p = 0.001) and previous year (2019). | 4 |
| Werneck et al. (2020)^150^ | Brazil | Changes in the clustering of unhealthy movement behaviors during the COVID-19 quarantine and the association with mental health indicators among Brazilian adults | Cross-sectional study | Analyze the prevalence of unhealthy movement behavior clusters before and during the COVID-19 pandemic, as well as to investigate whether changes in the number of unhealthy behaviors during the COVID-19 pandemic quarantine were associated with mental health indicators. | 38,353 adults | Considering the correlates, a greater increase in the prevalence of clustering unhealthy movement behaviors occurred among younger adults. Inactive + high TV (≥60, before: 7.2%; during: 10.5%), inactive + high PC (≥60, before: 21,4%; during: 28.4%), high TV + high PC (≥60, before: 2.3%; during: 2.9%), inactive + high TV + high PC (≥60, before: 8.3%; during: 25.0%). | 4 |
| Whatley et al. (2020)^68^ | United States | Younger and Older Adults’ Mood and Expectations Regarding Aging During COVID-19 | Study 1: Longitudinal study  Study 2: Cross-sectional study | Study 1: Investigate older adults’ mood and expectations regarding aging before and during the global pandemic. Study 2: Examine age differences in mood, expectations regarding aging, COVID-19 attitudes, and loneliness using a cross-sectional approach. | Study 1: n=49, mean age= 73.9 ± 7.5 (range 61-90)  Study 2: n=115 older adults, mean age= 69.7 ± 6.16 (range 60-90) | Study 1: They were maintaining positive mood and expectations about aging during the COVID-19 pandemic, but they reported more negative arousal. Study 2: Older adults reported more positive mood and higher expectations about aging than younger adults, supporting the finding that older adults, in general, were maintaining positivity following the onset of the COVID-19 pandemic. In both younger and older adults, more frequent feelings of loneliness were related to lower expectations regarding aging and more unpleasant mood. Older adults expressed greater concern for COVID-19. | Study 1: 2b  Study 2: 4 |
| Whitehead (2020)^69^ | United States | COVID-19 as a Stressor: Pandemic Expectations, Perceived Stress, and Negative Affect in Older Adults | Cross-sectional study | Investigate the association of older adults’ expectations concerning the pandemic’s duration and impact with perceived stress (PS) and negative affect (NA). | 714 residents of the United States and aged 60 and older, 63.2% being 60–69, 31.2% being 70–79, and 2.6% being 80 or older | Older adults’ expectations about COVID-19 (income decline, COVID duration, COVID long-term impact) at the early point in the pandemic at which this survey was taken were significantly associated with the amount of PS they were experiencing at the time, which in turn was associated with their level of NA. | 4 |
| Whitehead et al. (2020)^70^ | United States | Older Adults’ Experience of the COVID-19 Pandemic: A Mixed-Methods Analysis of Stresses and Joys | Cross-sectional study | Explore older adults’ reports of what about the pandemic is stressful, and what brings joy and comfort in the midst of stress | 825 U.S. adults aged 60 and older; 63.8% were 60-69, 30.7% were 70-79, and 5.5% were 80+ | The most commonly reported stressors were confinement/restrictions, concern for others, and isolation/loneliness; the most commonly reported sources of joy/comfort were family/friend relationships, digital social contact, and hobbies. Stress from concern for others, the unknown future, and contracting the virus to be significantly associated with poorer psychological well-being; faith, exercise/self-care, and nature were associated with more positive psychological well-being. | 4 |
| Wong et al. (2020)^84^ | China | Impact of COVID-19 on loneliness, mental health, and health service utilisation: a prospective cohort study of older adults with multimorbidity in primary care | Prospective cohort study | Describe changes in loneliness, mental health problems, and attendance to scheduled medical care before and after the onset of the COVID-19 pandemic. | 583 adults aged 60 years old and older (mean age: 70.9±6.1) | Compared with baseline scores before the COVID-19 outbreak, overall, social, and emotional loneliness were worse since the COVID-19 outbreak (P<0.05). For secondary outcomes, both anxiety (P = 0.011) and insomnia (P = 0.006) levels increased significantly. There was no significant change in depressive symptoms. Finally, a higher proportion of patients missed medical appointment (22% peri-COVID-19 vs. 16.5% pre-COVID-19; p<0.014). | 2b |
| Yamada et al. (2020)^142^ | Japan | Effect of the COVID-19 Epidemic on Physical Activity in Community-Dwelling Older Adults in Japan: A Cross-Sectional Online Survey | Cross-sectional study | Investigate changes in physical activity (PA) between January (before the COVID-19 epidemic) and April (during the COVID-19 epidemic) 2020 in community-dwelling older adults in Japan. | N=1,600, 65 to 84 years old without COVID-19, 74.0±5.6 years | Total PA time per week for older adults decreased by 65 minutes (−26.5%) from January to April 2020 (significantly decreased for all frailty categories). The total PA time in January and April 2020 was 330 and 210 minutes (−36.4) in robust older adults, 270 and 180 minutes (−33.3%) in pre-frail older adults, and 123 and 85 minutes (−30.9%) in frail older adults, respectively. | 4 |
| Younger et al. (2020)^130^ | United Kingdom | Health‐related quality of life and experiences of sarcoma patients during the COVID‐19 pandemic | Cross-sectional study | Investigate care experiences and health-related quality of life (HRQoL) in sarcoma patients during the COVID-19 pandemic. | N=350, range 16–92, 56.1±17.3 years | Fewer participants aged ≥65 years or older were categorized as lonely compared with participants 16-39 years (15% vs. 33%, respectively, p=0.004; UCLA abbreviated Loneliness Scale). Participants aged 65 years or older were the most likely to have postponed appointments among age groups (43% having postponed appointments; p=0.016) (43%). Loneliness was associated with younger age; adolescents and young adults (aged 16–39 years; 33%), middle-aged (aged 40–64; 29%), elderly (aged ≥ 65 years; 15%). | 4 |
| Zhang et al. (2020)^85^ | China | Status and influential factors of anxiety depression and insomnia symptoms in the work resumption period of COVID-19 epidemic: A multicenter cross-sectional study | Cross-sectional study | Analyze the status of anxiety, depression, and insomnia symptoms and influential factors in the work resumption period of COVID-19 epidemic. | N=3237, median = 37 years old, range 11–83 years; >64: 427 (13.2%) | Aged >64 years was an independent risk factors of depression symptoms, but not for anxiety symptoms (p=0.08) and for insomnia symptoms (p=0.06). Compared with participants younger than 18 years, participants older than 64 years reported higher odds of anxiety (OR: 1.60, 95%CI 0.91 to 2.87; GAD-7 scale), higher odds of depression symptoms (OR: 1.79, 95%CI 1.19 to 2.55; PHQ-9 scale) and higher odds of insomnia (OR: 1.83, 95%CI 0.97 to 2.92; ISI scale). | 4 |
| Zhao et al. (2020)^86^ | China | Mental health crisis under COVID-19 pandemic in Hong Kong, China | Cross-sectional study | To compare the mental health burden before and during the COVID-19 outbreak and identify the vulnerable groups by sociodemographic factors. | 2016 (N = 4036), 2017 (N = 4051) and the COVID-19 Health Information Survey (CoVHInS) in April 2020 (N = 1501) | Respondents who were older (aged 60+) tend to show a larger increase in all mental health outcomes. The increases in stress levels were significantly larger among older respondents. | 4 |
| Ziliak (2020)^73^ | United States | Food Hardship during the Covid-19 Pandemic and Great Recession | Cross-sectional survey | Assess how food hardship in the population overall and among seniors compares in the current crisis to the two decades preceding the Covid-19 pandemic. | Individuals aged 60 years and older. pre-COVID period: 2001 to 2019 December Current Population Survey; during COVID: Census Bureau’s Household Pulse Survey | Food insufficiency increased from 2.8% (December 2019; pre-COVID) to 4.9% (July 2020; during COVID) among individuals aged 60 years and older. Of note, this increase was smaller than the one observed when considering all adults aged 18 years and older (from 3.4% in December 2019 to 10.8% in July 2020). | 4 |
| Zipprich et al. (2020)^36^ | Germany | Knowledge, attitudes, practices, and burden during the covid-19 pandemic in people with Parkinson’s disease in Germany | Cross-sectional study | Explore knowledge, attitudes, practices, and burden in order to elucidate nonadherence to preventive measures. | 99 patients with Parkinson’s disease (PD) PD older (M age (years)=78.0) and PD younger (M age (years)=65.5)  And 21 controls (M age (years)=68.0) | Changes in behaviour were reported by 73 patients (99% performed at least one specific preventive behavior, and 86.9% have reduced social contacts and stayed home). 27.3% of patients continued to meet relatives face-to-face almost daily. Anxiety and worries about the current situation were reported by 58.6% of patients; 31.3% complained about a decrease in their mobility since the beginning of the restrictions, mainly because of worsening of PD and because regular therapies (e.g., physiotherapy) were canceled. | 4 |
